# Phlorotannin-Rich *Ascophyllum nodosum* Seaweed Extract Inhibits Influenza Infection

**DOI:** 10.3390/v16121919

**Published:** 2024-12-15

**Authors:** Daniele F. Mega, Parul Sharma, Anja Kipar, Udo Hetzel, Chloe Bramwell, Alan Merritt, Samuel Wright, Chris Plummer, Richard A. Urbanowicz, James P. Stewart

**Affiliations:** 1 Institute of Infection, Veterinary and Ecological Sciences, University of Liverpool, Liverpool L3 5RF, UK; d.f.mega@liverpool.ac.uk (D.F.M.); parul13@liverpool.ac.uk (P.S.); anja.kipar@uzh.ch (A.K.); c.bramwell@liverpool.ac.uk (C.B.); richard.urbanowicz@liverpool.ac.uk (R.A.U.); 2 Laboratory for Animal Model Pathology, Institute of Veterinary Pathology, University of Zurich, 8057 Zürich, Switzerland; udo.hetzel@uzh.ch; 3 Byotrol Technology Limited, Thornton Science Park, Chester CH2 4NU, UKswright@byotrol.com (S.W.); cplummer@byotrol.com (C.P.)

**Keywords:** influenza A virus, antiviral, seaweed, *Ascophyllum nodosum*

## Abstract

Seaweed-derived compounds are a renewable resource utilised in the manufacturing and food industry. This study focuses on an enriched seaweed extract (ESE) isolated from *Ascophyllum nodosum.* The ESE was screened for antiviral activity by plaque reduction assays against influenza A/Puerto Rico/8/1934 H1N1 (PR8), A/X-31 H3N2 (X31) and A/England/195/2009 H1N1 (Eng195), resulting in the complete inhibition of infection. Time of addition assays and FACS analysis were used to help determine the modes of action. The therapeutic potential of ESE was then explored using differentiated human bronchiole epithelial cells at the air–liquid interphase and a murine model challenged with IAV. The data indicates that ESE primarily interacts directly with virions, reducing mean virus–cell binding by 79.3% with 0.01 mg/mL ESE. Interestingly, ESE also inhibits the early and late stages of the influenza A lifecycle when treatment occurs after cell binding. This inhibitory effect appears to reduce the internalisation of the virus and the release of progeny virus by targeting neuraminidase activity, with IC50 values of 0.5 μg/mL for X31, 3.2 μg/mL for Eng195 and 12.8 μg/mL for PR8. The intranasal administration of 5 mg/kg ESE in mice infected with IAV reduced the viral load in lung tissue. ESE may be a promising broad-acting antiviral agent in the treatment of influenza infections.

## 1. Introduction

Influenza viruses are segmented single-stranded negative-sense RNA (-ssRNA) viruses in the *Orthomyxoviridae* family which are classified into four genera, Influenza A–D, and cause respiratory infections [1]. Influenza A viruses (IAV) are responsible for the majority of influenza infections in humans and five pandemics since 1889, the most recent of which was in 2009 and the most devastating in 1918 with over 50 million recorded deaths worldwide [2,3]. IAVs are further categorised into subtypes by their surface glycoproteins, hemagglutinin (HA; H1-H18) and neuraminidase (NA; N1-N11), of which A/H1N1 and A/H3N2 currently cause seasonal epidemics in humans [4]. Influenza viruses are constantly evolving due to genetic reassortment and low-fidelity RNA polymerase proofreading capabilities, which results in antigenic drift due to sequence changes encoding HA and NA, allowing escape from previously protective immune responses [5,6].

There are currently several licensed antivirals and seasonal vaccines available. One such antiviral, Amantadine, is directed at the viral M2 protein, targeting viral uncoating and the release of infectious nucleic acids [7,8]. The most commonly prescribed antivirals for influenza infections are neuraminidase inhibitors (NAIs) including Zanamivir and Oseltamivir (Tamiflu), which bind to the viral NA protein, blocking enzymatic function and therefore inhibiting the release of progeny virions [9], [10,11,12]. Resistance, however, has been reported for all currently licenced antivirals with Amantadine no longer recommended for the treatment of IAV infections [13,14,15]. This highlights the need to develop novel broad-acting antivirals, which could provide an alternative to current treatments or be used in combination with licenced antivirals.

Seaweed-derived compounds have been shown to have antiviral, anti-inflammatory and immune-modulating activities [16,17]. Fucoidans are sulphated carbohydrates found in brown algae that exhibit efficacy against IAV, binding surface glycoproteins inhibiting infection as well as the cellular EGFR pathway and viral neuraminidase activity in vitro [18]. Carrageenans are sulphated polysaccharides produced from red seaweed and have been well-studied for antiviral activity [19]. Iota-carrageenan displays antiviral activity against rhinovirus and IAV, most likely through the inhibition of cell binding or entry [20,21].

*Ascophyllum nodosum* is an edible species of brown algae commonly known as rockweed or knotted wrack found around the coast of the UK, western Europe and North America. It is rich in a variety of bioactive compounds including phenolic compounds such as phlorotannins and a range of polysaccharides including mannitol, laminarin and fucoidan [22]. Phlorotannin-rich extracts from *Ascophyluum nodosum* have been shown to have a range of anti-inflammatory, antioxidant and anti-ageing properties, while phlorotannins from other species exhibit antibacterial and even antiviral properties [23,24,25]. Given the range and diversity of these natural compounds, phlorotannins may be an attractive target for antiviral drug development.

This study focuses on the effect of a phlorotannin-rich enriched seaweed extract (ESE) isolated from the brown seaweed *Ascophyllum nodosum* [26] against IAV infection in vitro and in a murine model.

## 2. Materials and Methods

### 2.1. Seaweed Extract

Enriched Seaweed Extract (ESE) was produced by the hydroethanolic extraction of fresh *Ascophyllum nodosum*, harvested by a professional licensed harvester in L’Armor Pleubian, latitude 48,863°, longitude −3057°, followed by enrichment using C_18_ solid phase extraction. This afforded a phlorotannin-rich extract as previously described and characterised by LC-MS [26]. ESE was dissolved in phosphate-buffered saline (PBS) to a final concentration of 10 mg/mL. This stock solution was sterile filtered through a 0.2 μM filter and stored at 4 °C prior to use. The phenolic content was calculated using the Folin–Ciocalteu method, using phloroglucinol as a standard reagent. Briefly, 50% Folin–Ciocalteau reagent was added to a serially diluted standard or sample and mixed prior to the addition of saturated sodium carbonate solution. These solutions were incubated at RT for one hour prior, transferred to a 96-well plate and absorbance was read at 750 nm. The results were given as mg of phloroglucinol equivalents (PGE)/g of dry extract.

### 2.2. Virus, Cell Lines and Media

Madin Darby Canine kidney (MDCK) and MDCK-SIAT1 cells were maintained in DMEM supplemented with 10% foetal bovine serum (FBS) at 37 °C in 5% CO_2_. MDCK-SIAT1 cells were further supplemented with 1 mg/mL geneticin (G418). Influenza A virus A/Puerto Rico/8/1934 H1N1 (PR8), Influenza A virus A/X-31 H3N2 (X31) and Influenza A virus A/England/195/2009 H1N1 (Eng195) were propagated on MDCK or MDCK-SIAT1 cells.

### 2.3. Plaque Reduction Assays

IAV strains (PR8, X31, Eng195) were diluted to 25–50 PFU. For the cell pre-treatment assays, cells were treated with ESE diluted in serum-free DMEM supplemented with TPCK-trypsin (infection media) for one hour. ESE was removed and the cells were washed with PBS. The virus was added for one hour to allow for internalisation prior to the addition of an overlay. For virus pre-treatment assays, the virus was incubated 1:1 with ESE or infection media prior to the infection of cell monolayers for one hour. Post-entry events were investigated with the addition of ESE to plaque assay overlays 1 h post-infection of cell monolayers. Plaques were given as a percentage of an untreated control.

### 2.4. Growth Curves

Cells were grown to 80% confluency in 12-well plates and washed twice with PBS prior to infection MOI 0.001 for one hour at 37 °C in 5% CO_2_. The virus inoculum was removed and infection media or infection media containing ESE was added. The virus supernatant was harvested at the indicated time points and titrated by plaque assay on cell monolayers

### 2.5. Cell Viability Assays

Cells were grown to 80% confluency in 96-well plates and infected MOI 0.001 for one hour. The virus inoculum was removed and infection media containing ESE was added. Cell viability was measured by MTS assay (Promega, Madison, WI, USA) 72 h post-infection and given as a percentage of the untreated control.

### 2.6. Time of Addition Assays

MDCK cells were chilled to 4 degrees for 90 min and infected with IAV X31 MOI 0.01 for one hour on ice to allow virus binding. The temperature was then increased to 37 degrees to allow synchronised entry, and 0.01 mg/mL ESE was added at the indicated time points. At 12 h post-infection, the supernatant was collected and cells were lysed with TRIzol. The viral load in cells was measured by qRT-PCR of the IAV M gene and the titre was calculated by plaque assay.

### 2.7. IAV Nucleoprotein Localisation

MDCK cells were grown to 80% confluency and chilled at 4 degrees for 90 min on coverslips. X31 at an MOI of 3 was added to cells one hour prior at 4 degrees in the presence of 1 mM cycloheximide (Abcam, Cambridge, UK). Unbound bound virus was then removed with 3 washes of ice-cold PBS prior to the addition of infection media, 20 mM NH_4_ or 0.01 mg/mL ESE in the presence of 1 mM cycloheximide. Cells were rapidly warmed to 37 °C to allow synchronised entry in 5% CO_2_ for 3 h prior to fixing and permeabilisation with 100% methanol. IAV NP was detected with a mouse anti-NP (Abcam) and an Alexa Fluor 488-conjugated goat anti-mouse secondary antibody. The coverslips were mounted onto slides using ProLong™ Gold Antifade Mountant with DAPI (Thermofisher, Waltham, MA, USA) and imaged in lattice SIM mode using a Zeiss Elyra 7. Five fields of view were chosen at random consisting of over 100 cells. Images were SIM^2^ post-processed to generate super-resolved and widefield images.

### 2.8. Neuraminidase Inhibition Assay

Virus stocks were titred by a neuraminidase (NA) activity assay (Applied Biosystems, Foster City, CA, USA, 4457091) based on the NA activity of 5 μM 4-MU/60 min at 37 degrees. NA inhibition was calculated using a MUNANA-based neuraminidase assay (Applied Biosystems, 4457091). ESE or Zanamivir (Sigma, Darmstadt, Germany) was serially diluted and incubated with the virus for 30 min prior to the addition of NA–Fluor substrate for 60 min. The plate was read using an excitation wavelength range of 350 nm to 365 nm and an emission wavelength of 440 nm to 460 nm. IC_50_ values were calculated using sigmoidal curve fitting.

### 2.9. Animal Work

Animal work was reviewed and approved by the local University of Liverpool Animal Welfare Committee and performed under UK Home Office Project Licences PP4715265. Mice were all specified pathogen-free and maintained under barrier conditions in individually ventilated cages. Six- to eight-week-old female C57BL/6J mice were purchased from Charles River (Margate, UK). Mice were randomly assigned into cohorts.

For the administration of ESE, mice were lightly anaesthetised with isoflurane and 5 mg/kg ESE in PBS was administered intranasally.

For the ESE tolerability study, two cohorts of 4 mice each were used; cohort 1 received one dose of ESE in 50 μL PBS at day 1 while cohort 2 received 5 doses of ESE in 10 μL PBS on 5 consecutive days; animals were euthanised at day 5 by cervical dislocation. They were dissected immediately after death and samples from all major organs and tissues were collected and fixed in 10% buffered formalin.

For the infection study, 5 cohorts of 6 mice were used. Vehicle or mock-infected mice received 10 µL of PBS. The administration of 5 mg/kg ESE in 10 μL PBS started at different time points depending on the cohort. For the prophylactic cohort, treatment started 2 h prior to infection; for the time of infection cohort, ESE was administered together with the virus; and for the therapeutic cohort, treatment started at 3 hpi. PBS treatment for the vehicle and mock-infected mice started at 3 hpi. All prophylactic and time of infection cohort mice were treated again at 3 hpi, and all mice were treated daily thereafter.

For virus infection, mice were anaesthetised lightly with KETASET i.m. and challenged intranasally with 10^3^ PFU IAV X31 in 10 μL sterile PBS or were mock-infected with the same volume of PBS. Mice were sacrificed at day 5 post-infection. Lungs were removed immediately and the right lung was snap-frozen prior to downstream processing for virology. The left lung was fixed in 10% buffered formalin for 48 h. For histological and immunohistological examination, the lungs were routinely paraffin-wax-embedded.

### 2.10. Lung Homogenisation, RNA Extraction and DNase Treatment

The upper lobe and the lower lobe of the right lung were homogenised in 1ml TRIzol reagent or PBS for RNA extraction or titration by plaque assay, respectively. Tissues were homogenised using stainless steel beads and a TissueLyser (Qiagen, Hilden, Germany). Cell culture experiments were lysed in 0.5 mL of TRIzol reagent. The homogenates were clarified by centrifugation at 12,000× *g* for 5 min before full RNA extraction was carried out according to the manufacturer’s instructions. RNA was quantified and quality assessed using a Nanodrop (Thermofisher) before DNase treatment using the TURBO DNA-free™ Kit (Thermofisher) as per the manufacturer’s instructions.

### 2.11. qRT-PCR for Viral Load

Viral loads were quantified as previously described [27] using the GoTaq^®^ Probe 1-Step RT-qPCR System (Promega, Madison, Wisconsin, USA). The IAV primers and probe sequences are published as part of the CDC IAV detection kit using the following primers F: GACCAATCCTGTCACCTCTGAC and R: AGGGCATTTTGGACAAAGCGTCTA and probe: 56-FAM CGTGCCCAGTGAGCAAGGACTGCA 3IABkFQ. The IAV reverse genetics plasmid encoding the M gene was used to serve as a standard curve. The thermal cycling conditions for all qRT-PCR reactions were as follows: 1 cycle of 45 °C for 15 min and 1 cycle of 95 °C followed by 40 cycles of 95 °C for 15 sec and 60 °C for 1 min. The viral load was normalised relative to 18S rRNA. The 18S standard was generated by the amplification of a fragment of the murine 18S cDNA using the primers F: ACCTGGTTGATCCTGCCAGGTAGC and R: AGC CAT TCG CAG TTT TGT AC prior to purification using a QIAquick gel extraction kit (Qiagen). ISG15 gene expression was quantified using an SYBR Green-based real-time RT-PCR kit (Qiagen) and ISG15 QuantiTect primers (Qiagen, Hilden, Germany QT00322749) and normalised to 18S rRNA using 18S QuantiTect primers (Qiagen, QT02448075).

### 2.12. Infection of HBEC3-KT Cells Grown at Air–Liquid Interface

Immortalised human bronchial epithelial cells (HBEC3-KT) were grown on 12 mm transwells with 0.4 µm pore inserts (StemCell, Vancouver, BC, Canada) in complete PneumaCult Ex Pluis media (StemCell) until confluent. Cells were then air-lifted and basal media were replaced with complete PneumaCult ALI medium (StemCell) for 14 to 21 days. Differentiation was confirmed by the RT-PCR of differentiation markers. Differentiated cells were infected at the apical surface with 10^5^ PFU IAV Eng195. The inoculum was removed and the cells were washed three times with PBS. PBS or 0.01 mg/mL ESE treatment occurred at the indicated time points. At 24 and 48 h, apical surfaces were washed with PBS for 30 min and the virus titre was calculated by plaque assay.

### 2.13. SDS-PAGE Analysis of ESE-Treated IAV

Gradient-purified PR8 or concentrations of BSA were mixed with PBS or ESE to a final concentration of 2.5 mg/mL. The samples were further mixed with an equal volume of 2 × SDS sample buffer (0.125 M Tris-HCl [pH 6.8], 4% sodium dodecyl sulphate (SDS), 20% glycerol, 0.004% of bromophenol blue and 10% β-mercaptoethanol) and kept at 95 °C for 5 min. Proteins were analysed by 12% SDS–polyacrylamide gel electrophoresis (PAGE) prior to Coomassie R-250 or silver staining (Thermofisher).

### 2.14. Virus Labelling and FACS Analysis of Virus Binding

Gradient-purified IAV PR8 was labelled as previously described [28]. Briefly, the virus was labelled using an Alexa Fluor 488 fluorophore labelling kit (Thermofisher, A10235) and unreactive dye was removed by purification through a sucrose cushion. Infectious virus (PR8-488) was determined by plaque assay. For FACS analysis, 2 × 10^5^ MDCK cells were used per sample. Cells were detached with trypsin–EDTA and chilled to 4 degrees. PR8-488 was incubated with ESE or infection media for one hour prior to incubation with MDCK cells for one hour on ice. Unbound virus was removed by three washes with PBS and the cells were fixed with 4% PFA prior to FACS analysis using a BD FACSymphony A1 Flow Cytometer. Single cell populations were gated based on their forward and side scatter using FlowJo™ v10.10 Software.

### 2.15. Hemagglutination Assays

ESE was serially diluted two-fold in PBS. X31 or PR8 was also serially diluted two-fold and incubated 1:1 for 30 min with ESE in 96-well round-bottomed plates. Following incubation, 0.5% chicken red blood cells were added to each well and the HA titre was calculated after one hour.

### 2.16. Transmission Electron Microscopy (TEM)

The negative staining technique was applied. For this approach, purified PR8 was mixed with PBS or 2.5 mg/mL ESE (final concentration) for 30 min at room temperature, then fixed in 2% paraformaldehyde (PFA). A Formvar-coated EM grid was placed, with the Formvar side down, on a drop of virus solution for 1–3 min. The grid was removed, dabbed with filter paper and placed onto a drop of 2.0% phosphotungstic acid (PTA), pH 7.0, for 1 min. Subsequently, the specimen was dried and viewed under a Philips CM10 (FEI, Hillsboro, OR, USA), operated with a Gatan Orius Sc1000 digital camera (Gatan, Pleasanton, CA, USA, Microscopical Suite, Digital Micrograph, Version 3.30.2016.0).

### 2.17. Histological and Immunohistological Examination

For the histological examination, tissue samples were trimmed after 48 h of formalin fixation and subsequently stored in 70% ethanol until processing and routinely paraffin-wax-embedded. Sections (2–4 µm) from the tissues in the tolerability study and from left lungs in the infection experiment were prepared and routinely stained with haematoxylin eosin (HE) for histological examination. Consecutive sections from the lungs in the infection experiment were subjected to immunohistological staining for IAV antigen, using the horseradish peroxidase method, a goat anti-IAV (H1N1; virions) antibody (Meridian Life Sciences Inc., Memphis, TN, USA) and a previously published protocol [29].

### 2.18. Statistical Analysis

Data were analysed using the Prism package (Version 10.1.1). p-values were set at a 95% confidence interval. Statistical tests used are stated in figure legends. All differences not specifically stated to be significant were not significant (*p* > 0.05).

## 3. Results

### 3.1. Anti-Influenza Activity of Enriched Seaweed Extract (ESE)

**Figure 1:**
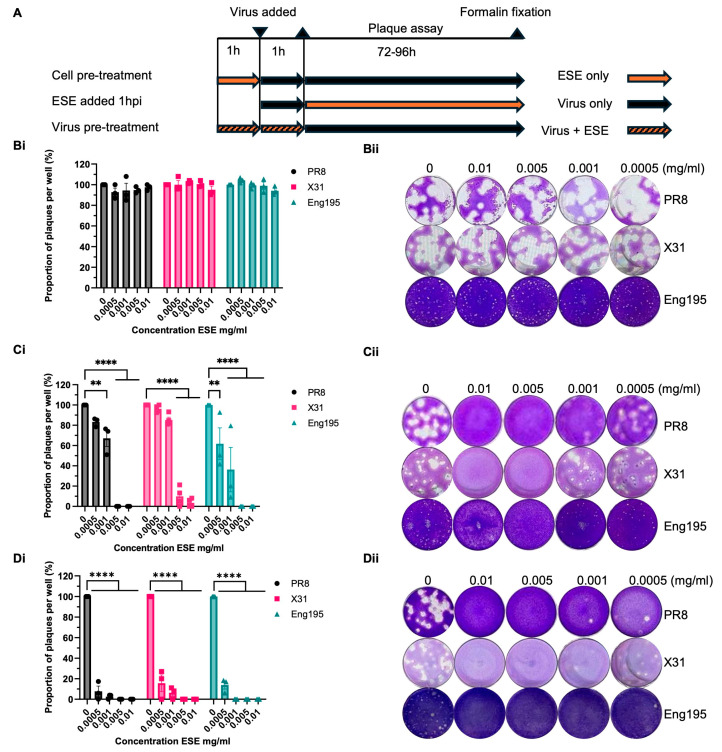
Plaque reduction assay IAV ESE. (**A**) Schematic of plaque reduction analysis. (**Bi**,**Bii**) MDCK cells pre-treated with ESE or infection media. Cells were pre-treated for one hour. ESE was then removed, and the cells were washed with PBS prior to infection with IAV PR8, X31 or Eng195. The inoculum was removed following 1 h of adsorption and a plaque assay overlay was added until plaque formation prior to fixing and staining. (**Ci**,**Cii**) ESE added following virus adsorption. MDCK monolayers were infected for one hour with IAV, the inoculum was removed and a plaque assay overlay containing ESE was added prior to fixing and staining upon plaque formation. (**Di**,**Dii**) IAV PR8, X31 or Eng195 were pre-treated with ESE for one hour prior to the infection of MDCK cell monolayers. The inoculum was removed following one hour of adsorption and a plaque assay overlay was added prior to fixing and staining upon plaque formation. Data are represented as the mean ± SEM of three or more independent experiments. Asterisks indicate statistical difference (two-way ANOVA with Dunnett’s multiple comparisons test; ** *p* < 0.01, **** *p* < 0.0001).

The ability of ESE to inhibit virus infection in cells was screened by plaque reduction assays using three strains of IAV. Influenza A virus A/Puerto Rico/8/1934 H1N1 (PR8), Influenza A virus A/X-31 H3N2 (X31) and Influenza A virus A/England/195/2009 H1N1 (Eng195) were used to represent different subtypes and a more recent pandemic strain. Non-cytotoxic concentrations of ESE were determined by measuring cell viability by MTS assay following 72 h of incubation with ESE, the longest time point used in the following experiments (Supplementary Figure S1). The mode of action was investigated by adding ESE one hour post-infection (hpi) to the plaque assay overlay or by either pre-treating the virus or cells for one hour prior to infection (Figure 1A). The number of plaques is given as a proportion of the untreated control. The pre-treatment of IAV (Figure 1Di,Dii) with ESE produced the greatest reduction in plaques in a dose-dependent manner, resulting in up to 100% reduction in all three strains tested at the highest concentrations. Pre-treating cells with ESE (Figure 1Bi) did not cause a significant reduction in plaques compared to the untreated control, suggesting that ESE does not interact with cellular receptors for virus binding. The addition of ESE at one hpi also resulted in a significant reduction in plaques (Figure 1Ci) in a dose-dependent manner against all three strains and was most effective against the Eng195 strain. These data suggest that non-cytotoxic concentrations of ESE most potently inhibit the early stages of the virus life cycle prior to virus cell entry while also potentially inhibiting events following cell entry.

### 3.2. ESE Interacts with Virions

**Figure 2:**
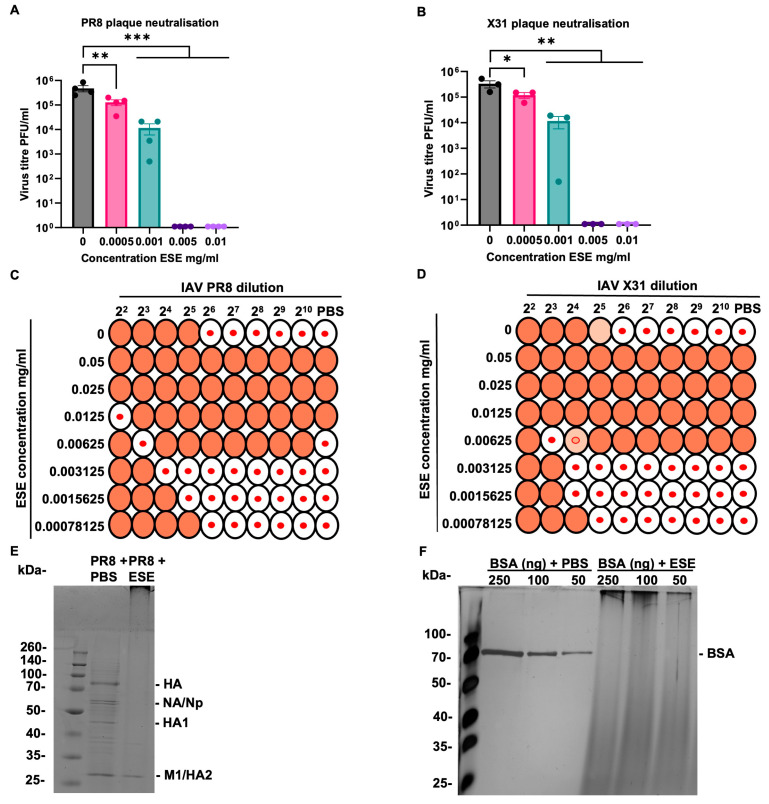
ESE interacts with hemagglutinin and causes protein aggregation. (**A**,**B**) Plaque neutralisation. PR8 (**A**) and X31 (**B**) were diluted to 10^6^ PFU/mL and incubated 1:1 with the shown concentrations of ESE or infection media for one hour. Serial ten-fold dilutions were then performed and used to infect MDCK monolayers for one hour prior to the addition of the overlay and fixing and staining 3 days later. Data are represented as the mean ± SEM of three or more independent experiments. Asterisks indicate statistical difference (one-way ANOVA with Dunnett’s multiple comparisons test; * *p* < 0.05, ** *p* < 0.01, *** *p* < 0.001). (**C**,**D**) HA assay representation. PR8 and X31 were serially diluted two-fold in PBS. ESE was also serially diluted two-fold and incubated in equal volumes with IAV for 30 min prior to the addition of 0.5% chicken RBC. HA titres were then calculated. (**E**,**F**) ESE causes the aggregation of proteins. PR8 (**E**) or shown concentrations of BSA (**F**) were incubated with PBS or 2.5 mg/mL ESE prior to separation on an SDS-PAGE gel and Coomassie (**E**) or silver (**F**) staining.

The plaque reduction assays displayed the greatest effect after the pre-treatment of the virus with ESE, suggesting a direct mode of action on the virion and the inhibition of virus cell entry. This was further explored using a higher starting concentration of virus followed by serial dilutions to assess whether inhibition could be reversed or diluted out. PR8 and X31 were diluted to 10^6^ PFU/mL and mixed 1:1 with ESE for one hour prior to serial dilutions onto cell monolayers. ESE neutralised both PR8 and X31 (Figure 2A,B) at 0.005 and 0.01 mg/mL while 0.001 mg/mL caused approximately one log reduction in titre against both PR8 and X31. The ability of ESE to neutralise high titres of IAV despite subsequent serial dilutions suggests a strong potentially virucidal interaction between the extract and the virus. The possibility of an interaction with a virus protein important for entry such as HA was investigated next. HA inhibition assay was performed for both PR8 and X31 displaying HA titres 2^5^ and 2^4^ respectively. ESE appeared to cause agglutination at high concentrations. An amount of 0.0125 mg/mL ESE inhibited agglutination at the lowest dilution of PR8; however, the remaining dilutions of the virus and PBS control still displayed agglutination. This may indicate that ESE interacted with the high titre of the virus at 2^2^ and was therefore unable to cause agglutination. An amount of 0.003125 mg/mL reduced the HA titre to 2^3^ for both PR8 and X31 while 0.0015625 mg/mL reduced HA titres to 2^4^ and 2^3^ for PR8 and X31 respectively (Figure 2C,D). The lowest concentration, 0.00078125 mg/mL, had no effect on HA titres for either PR8 or X31. ESE was therefore presumed to be binding viral proteins and this was analysed by separating purified IAV that had been incubated with ESE or PBS by SDS-PAGE. Coomassie staining showed that viral protein bands disappeared when incubated with ESE from their expected molecular weights and appeared to aggregate at the top of the gel despite denaturing (Figure 2E), something previously seen by plant-derived tannins [30]. The treatment of BSA with ESE also caused a similar outcome, with the protein band appearing at the top of the gel following silver staining (Figure 2F).

### 3.3. Virus–Cell Binding Is Inhibited by ESE

**Figure 3:**
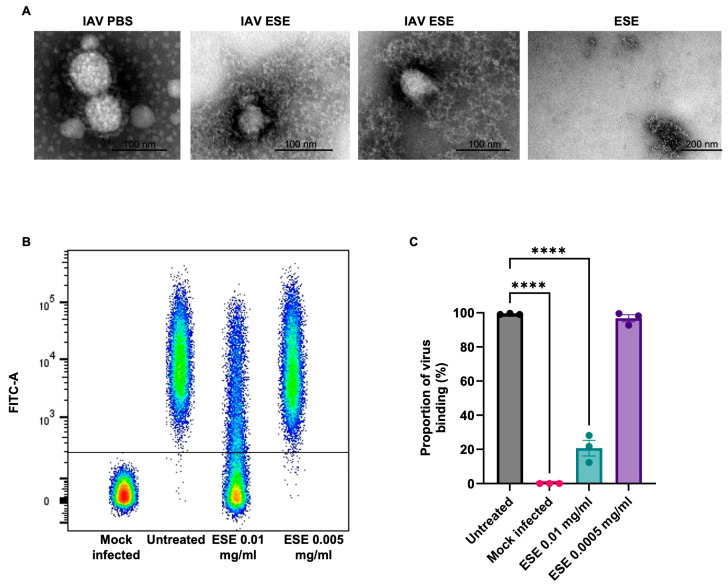
ESE interacts with IAV and prevents cell binding. (**A**) IAV PR8 in the presence of PBS or ESE, transmission electron microscopy, negative staining technique. IAV was purified through a sucrose cushion and mixed with PBS or 2.5 mg/mL final ESE and fixed with PFA. **Left**: IAV with PBS, showing an aggregate of regularly shaped virions of appr. 70 nm diameter. **Middle**: IAV with ESE. Virions are small (appr. 40 nm diameter) and appear partly fragmented. **Right**: ESE, without virions. (**B**,**C**) FACS analysis of PR8-488 binding to MDCK cells. PR8-488 MOI 3 was incubated with infection media or shown concentrations of ESE for one hour prior to the infection of MDCK cells for one hour at 4 degrees. The unbound virus was removed by PBS washing prior to fixation and analysis. (**B**) Dot plot representation of virus–cell binding. (**C**) Proportion of virus binding calculated by FACS analysis. Data are represented as the mean ± SEM of three independent experiments. Asterisks indicate statistical difference (one-way ANOVA with Dunnett’s multiple comparisons test; **** *p* < 0.0001).

The data so far suggest that ESE is binding to IAV, potentially preventing cell entry. This was further explored using transmission electron microscopy (TEM). Purified IAV PR8 was examined by TEM in the presence of ESE or PBS using the negative staining technique. Large numbers of regularly shaped, 70 nm sized virions were detected in the control (PBS) sample. In the presence of high concentrations of ESE, the virions were smaller, approximately 40 nm, and appeared partly fragmented (Figure 3A). This interaction is likely to inhibit cell binding, and this was further explored using IAV PR8 labelled with the fluorescent dye Alexa Fluor 488 (PR8-488). MDCK cells were chilled to 4 °C prior to infection with PR8-488 pre-treated with ESE or infection media to allow virus binding but not internalisation. FACS analysis of cells shows that the pre-treatment of PR8-488 with 0.01 mg/mL ESE caused a significant reduction (79.3% mean reduction) in virus–cell binding at 0.01 mg/mL (Figure 3C).

### 3.4. ESE Promotes Cell Survival and Reduces Virus Titre After Virus Cell Entry

**Figure 4:**
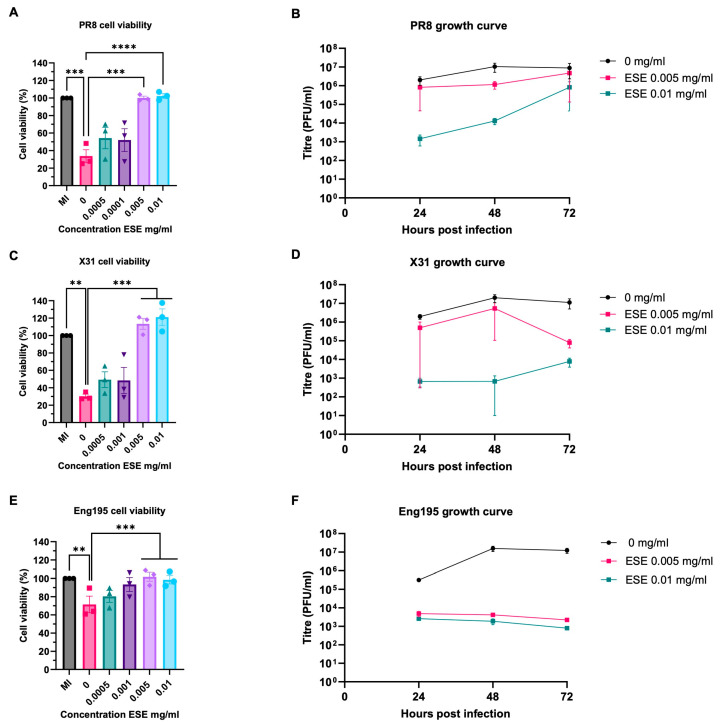
ESE promotes cell survival and reduces virus titre when added after infection. (**A**,**C**,**E**) MDCK cells were mock infected or infected with IAV PR8 (**A**), X31 (**C**) or Eng195 (**E**) MOI 0.001 for one hour. The inoculum was then removed and ESE concentrations or infection media were added. Cell viability was assessed by MTS assay 72 h post-infection and given as a percentage of a mock-infected control (n = 3). Data are represented as the mean ± SEM. Asterisks indicate statistical difference (two-way ANOVA with Dunnett’s multiple comparisons test; ** *p* < 0.01, *** *p* < 0.001 **** *p* < 0.0001). (**B**,**D**,**F**) IAV growth curves. MDCK cells were infected with IAV PR8 (**B**), X31 (**D**) or Eng195 (**F**) MOI 0.001 for one hour. The inoculum was then removed and ESE concentrations or infection media were added. The supernatant was harvested at the indicated time points and titrated by plaque assay on MDCK cell monolayers. Data represent the mean value ± SEM (n = 3).

The initial screening of anti-influenza activity elucidated another antiviral mechanism in events following virus–cell binding. This was investigated using the same three strains of IAV and the addition of ESE one hour after infection following cell entry. Cells were infected at a low MOI of 0.001 to allow for multiple rounds of infection. Viable cells were measured by MTS assay at 72 hpi and given as a percentage of a mock-infected control. ESE reduced virus-induced cell death in a dose-dependent manner in all three strains of IAV and no cytotoxicity was observed (Figure 4A,C,E). To assess whether the promotion of cell survival was due to a reduction in virus titre, growth curves were performed. An amount of 0.01 mg/mL of ESE reduced the virus titre in all three strains, while 0.005 mg/mL also resulted in a reduction in virus titre (Figure 4B,D,F). Interestingly, the PR8 titre following the addition of ESE appeared to increase over the 72 h and was a similar titre to the untreated control at 72 h, which was not seen for X31 or Eng195 strains.

### 3.5. Virus Release and Neuraminidase Activity Is Inhibited by ESE

**Figure 5:**
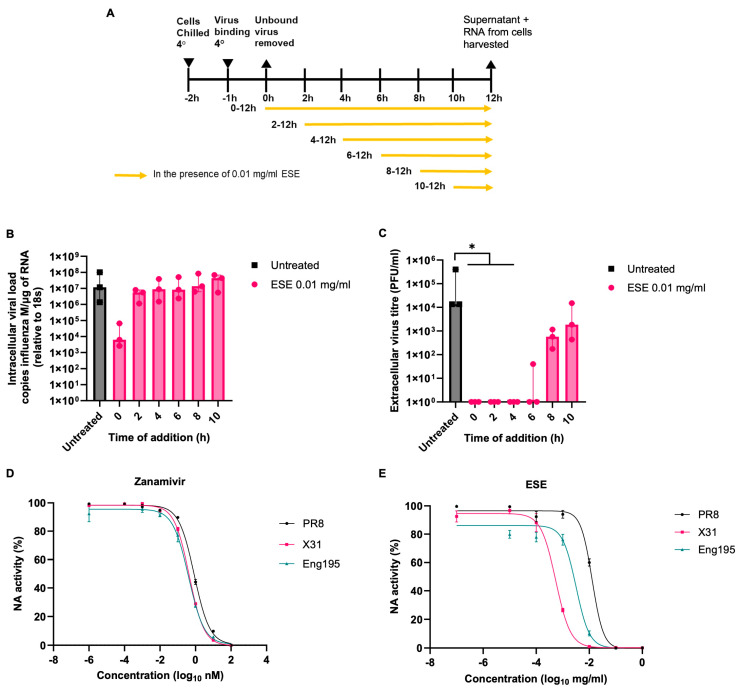
IAV release is inhibited by ESE. (**A**) Schematic of time of addition assay. MDCK cells were chilled to 4 degrees and infected IAV X31 MOI 0.01 for one hour on ice to allow virus binding. The temperature was then increased to 37 degrees to allow synchronised entry and 0.01 mg/mL ESE was added at the indicated time points. At 12 h post-infection, cells were lysed and the supernatant was collected. Viral load in cells (**B**) and titre in the supernatant (**C**) were measured by qRT-PCR and plaque assay, respectively. Data represent the median value + interquartile range (IQR) of three independent experiments. Asterisks indicate statistical difference (Kruskal–Wallis with Dunn’s multiple comparisons test; * *p* < 0.05). (**D**,**E**) Fluorescent neuraminidase substrate (MUNANA)-based neuraminidase inhibition assay using Zanamivir (**D**) or ESE (**E**) (n = 3).

Time of addition assay over one virus replication cycle (12 h) was carried out to assess which stage of the virus lifecycle was being affected by ESE. ESE appeared particularly effective at reducing IAV X31 virus titre and virus-induced cell death (Figure 4C,D). Therefore, MDCK cells were infected with X31 MOI 0.01 on ice to allow virus binding but not internalisation. Virus entry was synchronised by rapidly increasing the temperature to 37 degrees and 0.01 mg/mL ESE was added at different time points (0, 2, 4, 6, 8, 10 hpi) (Figure 5A). Virus supernatant was collected at 12 hpi and cells were lysed following the completion of one virus lifecycle (12 hpi). Intracellular virus titres (Figure 5B), calculated by measuring viral load in cell lysate at 12 hpi by qRT-PCR, were compared to extracellular virus titres in the supernatant at 12 hpi titrated by plaque assay (Figure 5C). Viral load at 12 hpi was reduced when ESE was added at 0 h following cell binding, although not significantly, while there was no noticeable decrease in viral load at 12 hpi when ESE was added at the remaining time points (Figure 5B). Interestingly, despite the high viral load, the release of infectious virus in the supernatant at 12 hpi was inhibited with the addition of ESE at 0, 2 and 4 h, while the titre was reduced with the addition of ESE at 6, 8 and 10 h (Figure 5C). Influenza viruses utilise NA activity to cleave sialic acid at the cell surface from progeny virus, facilitating virus release. Therefore, the ability of ESE to inhibit the NA activity of PR8, X31 and Eng195 was explored using the fluorescent neuraminidase substrate MUNANA, with Zanamivir, a known NA inhibitor, used as a positive control (Figure 5D,E). ESE inhibited the NA activity of all three strains of IAV, most effectively against X31 and least effectively against PR8. The IC50 values are shown in Table 1. These values may explain the increased virus titre of PR8 at 72 hpi compared to X31 and Eng195 (Figure 4B).

### 3.6. Nuclear Import and Internalisation of IAV Nucleoprotein Is Reduced

**Figure 6:**
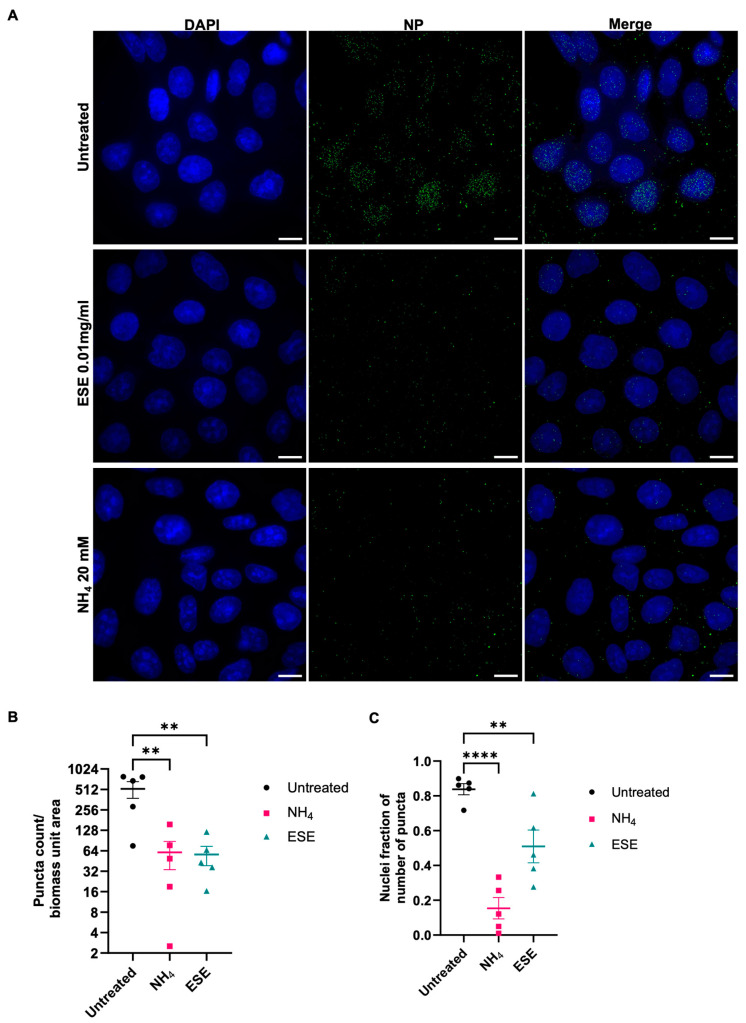
ESE reduces the internalisation of IAV NP. (**A**) MDCK cells were grown to 80% confluency and chilled at 4 degrees for 90 min. X31 at an MOI of 3 was added to cells for one hour at 4 degrees in the presence of 1 mM cycloheximide. Unbound bound virus was then removed with 3 washes of ice-cold PBS prior to the addition of infection media, 20 mM NH_4_ or 0.01 mg/mL ESE. Cells were rapidly warmed to 37 °C to allow synchronised entry in 5% CO_2_ for 3 h prior to fixing and permeabilisation with 100% methanol. IAV NP was detected with a mouse anti-NP and an Alexa Fluor 488-conjugated goat anti-mouse secondary antibody. Bars = 10 μM. (**B**) Puncta count and nuclei fraction (**C**) were calculated from 5 fields of view chosen at random representing over 100 cells. Data are represented as the mean ± SEM. Asterisks indicate statistical difference (one-way ANOVA with Dunnett’s multiple comparisons test; ** *p* < 0.01, **** *p* < 0.0001).

The reduction in viral load when ESE was added post-binding indicated a disruption of the virus lifecycle prior to replication. The localisation and internalisation of input IAV nucleoprotein (NP) was therefore explored in the presence of a protein synthesis inhibitor, cycloheximide. Briefly, MDCK cells were chilled to 4 °C to allow virus binding but not entry. IAV X31 was added at MOI 3 and allowed to bind for one hour. ESE or the endosome acidification inhibitor ammonium chloride (NH_4_) were then added, and virus entry was synchronised at 37 °C for 3 h. Cells were then fixed and stained for IAV nucleoprotein (NP). In the untreated sample, NP localised to the cell nucleus as expected while NH_4_ prevented the localisation of NP to the cell nucleus and reduced the number of puncta (Figure 6A). ESE also appeared to reduce internalisation with a decrease in puncta detected while the localisation of NP to the cell nucleus also appeared to be reduced compared to the untreated control (Figure 6B,C).

### 3.7. Investigating ESE Effectiveness on 3-D Model

**Figure 7:**
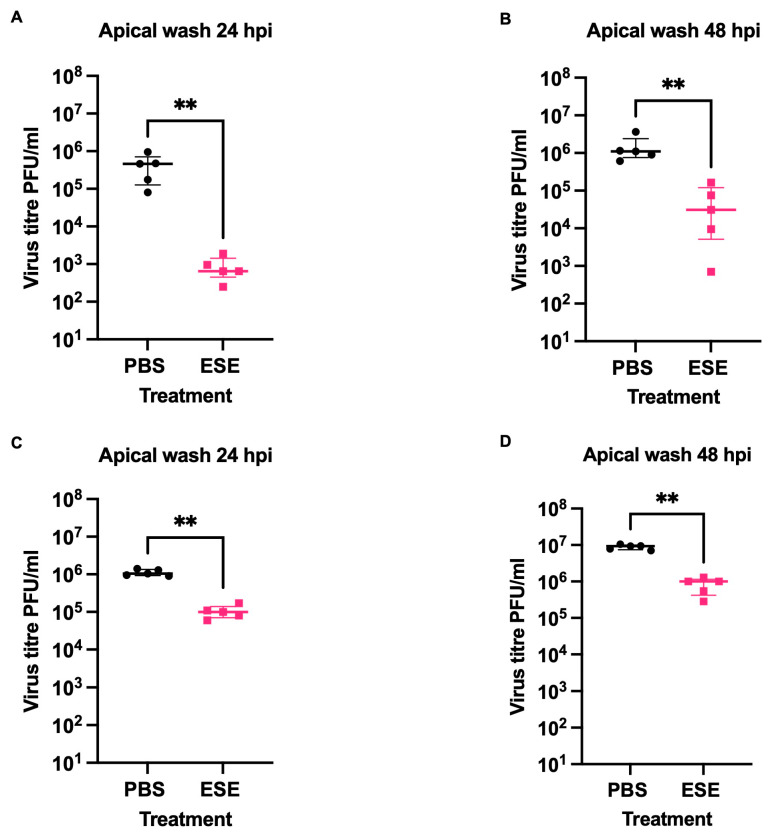
Investigating ESE effectiveness on a differentiated 3-D model. (**A**,**B**) HBEC3-KT cells were grown at the air–liquid interface for 14 to 21 days. Differentiation was confirmed by endpoint PCR. Cells were infected at the apical surface with 10^5^ PFU IAV ENG195 in the presence of 0.01 mg/mL ESE or PBS. Apical washes were performed at 24 (**A**) and 48 (**B**) hours and virus titre was calculated by plaque assay. (**C**,**D**) Differentiated HBEC3-KT cells were infected at the apical surface with 10^5^ PFU IAV ENG195. An amount of 0.01 mg/mL ESE or PBS was added to the apical surface at 2 hpi, 6 hpi, 24 hpi and 30 hpi. Apical washes were performed at 24 (**C**) and 48 (**D**) hours and virus titre was calculated by plaque assay. Data represent the median value + IQR of 5 biological replicates. Side-by-side comparisons were made using the Mann–Whitney U test (** represents *p* < 0.005).

The effectiveness of ESE in inhibiting IAV infection of human bronchial epithelial cells (HBEC3-KT) grown at the air–liquid interface was explored. Cells were allowed to differentiate for 14 to 21 days prior to infection, and differentiation was confirmed by the endpoint PCR of differentiation markers MUC5AC, CBE1, SCGB1A1, BPIFA1 and TEKT1. The inhibition of virus cell entry was investigated by infecting cells with 10^5^ PFU IAV Eng195 in the presence of ESE or PBS at the apical surface and the virus titre was calculated from apical washes at 24 and 48 h. A significant reduction in virus titre was observed at both 24 and 48 hpi compared to the PBS-treated control (Figure 7A,B). The potential of ESE to inhibit infection following virus entry was investigated next, with cells again being infected with 10^5^ PFU IAV Eng195 and ESE or PBS being dosed repeatedly at the apical surface at 2 hpi, 6 hpi, 24 hpi and 30 hpi. The virus titre was again calculated from apical washes at 24 and 48 hpi. A significant reduction in virus titre was observed at both 24 and 48 hpi compared to the PBS-treated control (Figure 7C,D).

### 3.8. Intranasal Administration of ESE Reduces Viral Load in Mice Infected with IAV

The effectiveness of ESE intranasal administration was examined in a mouse model. In the first step, we determined whether ESE had any pathological effect when administered intranasally. For this, 6–8-week-old female C57Bl/6J mice were treated intranasally with 5 mg/kg ESE, either as one dose in 50 µL PBS to ensure that a sufficient amount of ESE reached the lung parenchyma, i.e., the alveoli, or as five doses in 10 µL PBS on 5 consecutive days. All mice were euthanised at day 4 post-administration. Mice receiving ESE in one dose in 50 μL PBS exhibited mild weight loss at day 1 (Supplementary Figure S2) but then showed progressive weight gain. In contrast, mice receiving lower daily doses of 10 μL basically maintained their weights throughout the course of the experiment (Supplementary Figure S2). The post-mortem examination did not reveal any gross pathological changes in the animals. All relevant organs/tissues were subjected to a histological examination. This did not reveal any pathological changes in any organs apart from the lungs of the mice that had received ESE in one 50 µL dose. In all four mice, the lungs exhibited mild inflammatory changes, represented by focal granulomatous (i.e., macrophage-dominated) infiltrates; this likely represents a response to inhaled ESE (Supplementary Figure S3). These findings indicate that intranasal ESE application has no systemic pathological effect but show that ESE can induce a mild foreign body reaction in the lung when applied intranasally in a larger fluid volume. This is not seen when most of the ESE can be expected to remain in the upper respiratory tract, i.e., when applied in lower volumes. However, the inflammatory reaction was only mild and did not induce any clinical signs as all mice had gained weight by two days after instillation, which can be considered the shortest time span for a granulomatous reaction to have developed, since the uptake of suitable inhaled material by alveolar macrophages alone takes 6–12 h [31]. The results of the histological examination of the individual animals are provided in Supplementary Table S2.

**Figure 8:**
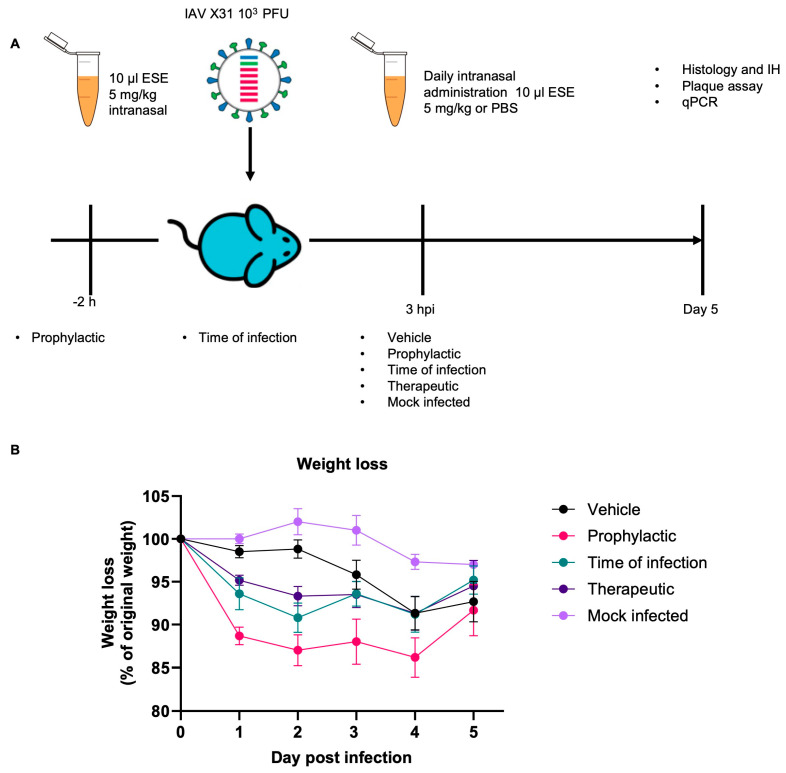
Intranasal administration of ESE in vivo. (**A**) Schematic of in vivo infection and treatment administration. Female C57BL/6 mice were challenged intranasally with 10^3^ PFU IAV X31 in 10 µL. Daily intranasal administration of 10 µL of 5mg/kg ESE or PBS started at the indicated time points and continued until day 5 when mice were sacrificed by cervical dislocation. (**B**). Mice were monitored for weight loss at the indicated time points (n = 6). Data represent the mean value ± SEM. Comparisons were made using a repeated measures two-way ANOVA (Supplementary Table S1).

For the subsequent infection experiment, ESE was applied at the above-mentioned dose, and in 10 µL PBS, to avoid any ESE-induced inflammatory reaction. Briefly, mice were infected intranasally with a sub-lethal dose of IAV X31. Three ESE treatment approaches were taken, prophylactical (treatment starting at 2 h pre-infection), at the time of infection, and therapeutical (treatment starting at 3 hpi); treatment then continued daily until sacrifice at day 5 (Figure 8A). The prophylactical cohort and time of infection cohort were also dosed again at 3 hpi. Control mice received daily doses of PBS starting at 3hpi.

Vehicle-treated infected animals showed the typical weight change after IAV infection, with obvious weight loss by day 3 and peaking at day 4 [27]. With ESE treatment, a drop in weight was observed at day 1 prior to virus-mediated weight loss which started at day 3; this was most intense in the prophylactic group, where it remained rather stable at the overall lowest level until day 4 (Figure 8B). This initial drop in weight might be the consequence of the repeated anaesthesia (three times) this group of mice was subjected to on day 0, as this weight loss was not seen previously when administering ESE (Supplementary Figure S2) and no clinical manifestations were observed. The trend was similar in the other two treatment groups, and at day 5, the weights of the various groups did not differ significantly (Supplementary Table S1).

**Figure 9:**
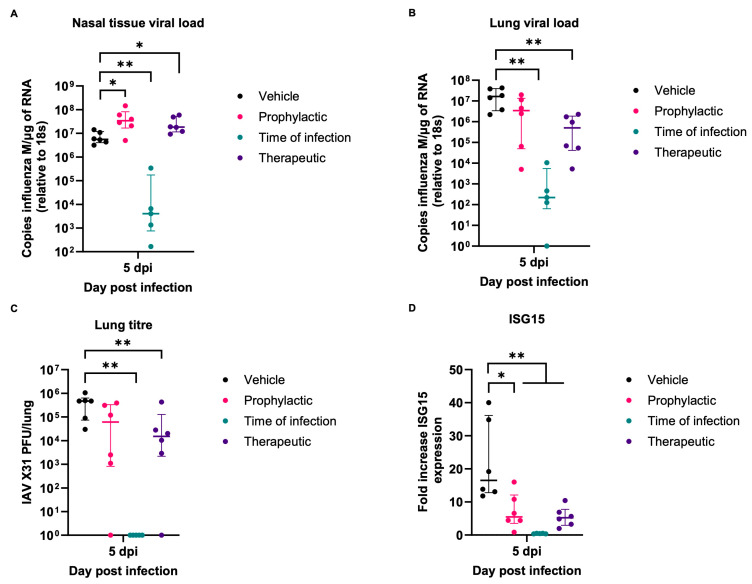
Viral load in mice challenged with IAV. Viral load in RNA extracted from nasal tissue (**A**) or lungs (**B**) was calculated by qRT-PCR of the influenza M gene and normalised relative to levels of 18S rRNA (n = 6). (**C**) IAV titre from the homogenised right lung lobe was calculated by titration on MDCK cell monolayers (n = 6). (**D**) ISG15 gene expression was calculated by qRT-PCR of lung RNA using the delta–delta ct method and given as a fold increase in mock-infected mice (n = 6). Data represent the median value + IQR. Side-by-side comparisons were made using the Mann–Whitney U test (* represents *p* < 0.05, ** represents *p* < 0.005).

Examination of the animals at the end of the experiment, i.e., day 5 post-infection, confirmed IAV infection in all mice. They all harboured viral RNA in the nasal tissue, as determined by qRT-PCR for the viral M gene (Figure 9A). Interestingly, the viral load was significantly higher in both the therapeutic (median 1.83 × 10^7^ copies of M/μg RNA) and prophylactic (median 3.43 × 10^7^ copies of M/μg RNA) treatment cohorts compared to the vehicle-only cohort (median 5.60 × 10^6^ copies of M/μg RNA), whereas it was significantly lower in mice for which the treatment had started at the time of infection (median 4.04 × 10^3^ copies of M/μg RNA). The latter group also exhibited the lowest viral loads (median 222 copies of M/μg RNA) in the lungs (Figure 9B), from which infectious virus could not be isolated when the virus titre was determined by plaque assay performed on homogenised lung tissue (Figure 9C). Significantly lower viral loads (median 5.05 × 10^5^ copies of M/μg RNA), as well as significantly lower virus titres (median 1.52 × 10^4^ PFU/lung), were also found in the lungs of mice that had received the therapeutic treatment compared to the vehicle (median 1.64 x 10^7^ copies of M/μg RNA and 4.70 × 10^5^ PFU/lung) controls (Figure 9B,C). The prophylactic treatment regime resulted in a reduction in viral loads (median 3.46 × 10^6^ copies of M/μg RNA) and in the virus titre in the lungs (median 6.13 × 10^4^ PFU/lung), although the difference to mice receiving the vehicle did not reach significance (*p* = 0.0584). It has been previously reported that seaweed-derived compounds can increase the interferon response to infection [18]. This was explored by analysing ISG15 expression. ISG15 expression in lungs (Figure 9D) was significantly lower in all cohorts compared to the vehicle-only cohort and is supportive of decreased infection in mice intranasally treated with ESE.

**Figure 10:**
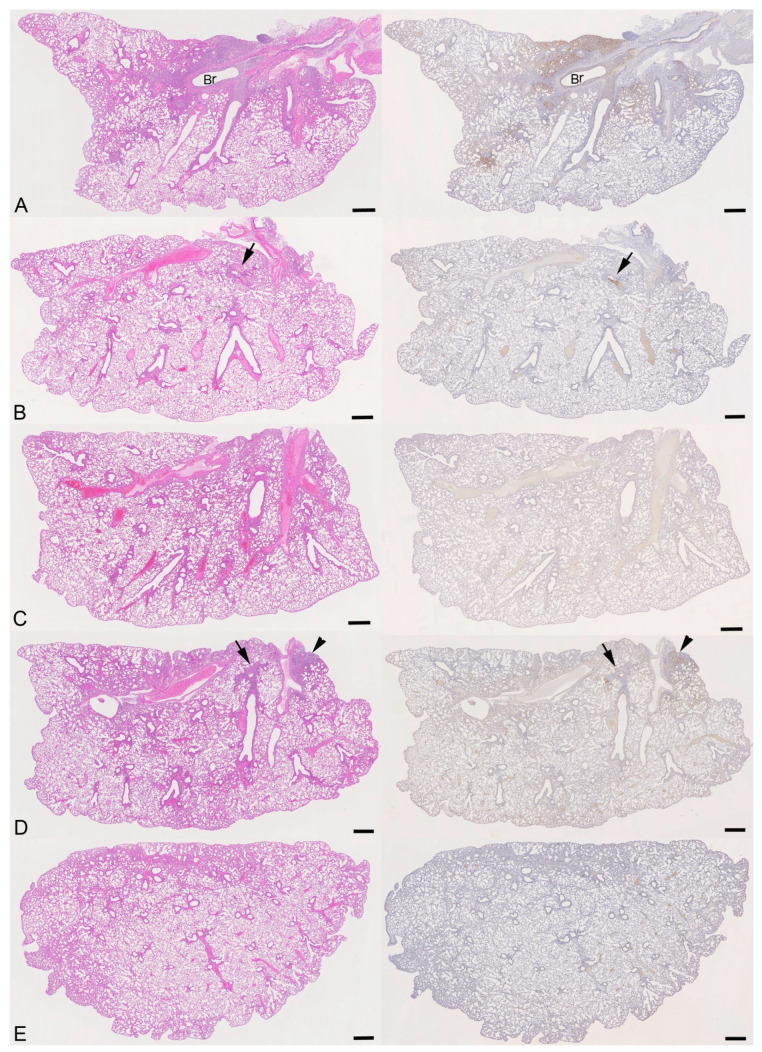
Histological features and viral antigen expression in mice challenged with IAV and treated with ESE. (**A**) Animal treated with PBS (#1.6). Necrotic bronchitis and bronchiolitis with extensive viral antigen expression and desquamative pneumonia in adjacent parenchymal areas (for higher magnification, see Figure 11A). Br: bronchus. (**B**) Animal of prophylactic treatment scheme (#2.6). Changes are restricted to one bronchiole (arrow) with a patch of infected, IAV antigen-positive epithelial cells and some material in the lumen (for higher magnification, see Figure 11B). (**C**) Animal of time of infection treatment scheme (#3.6). The lung parenchyma appears unaltered, and there is no evidence of viral antigen expression. (**D**) Animal of therapeutic treatment scheme (#4.6). Focal area with peribronchial leukocyte infiltration and small bronchioles with infected epithelial cells (arrow) and focal parenchymal area with changes consistent with desquamative pneumonia and viral antigen expression in numerous alveolar epithelial cells and macrophages (arrowhead). (**E**) Mock-infected control animal treated with PBS (#5.2). The lung parenchyma is unaltered, and there is no evidence of viral antigen expression. Left column: HE stain; right column: immunohistology, hematoxylin counterstain. Bars = 500 µm.

**Figure 11:**
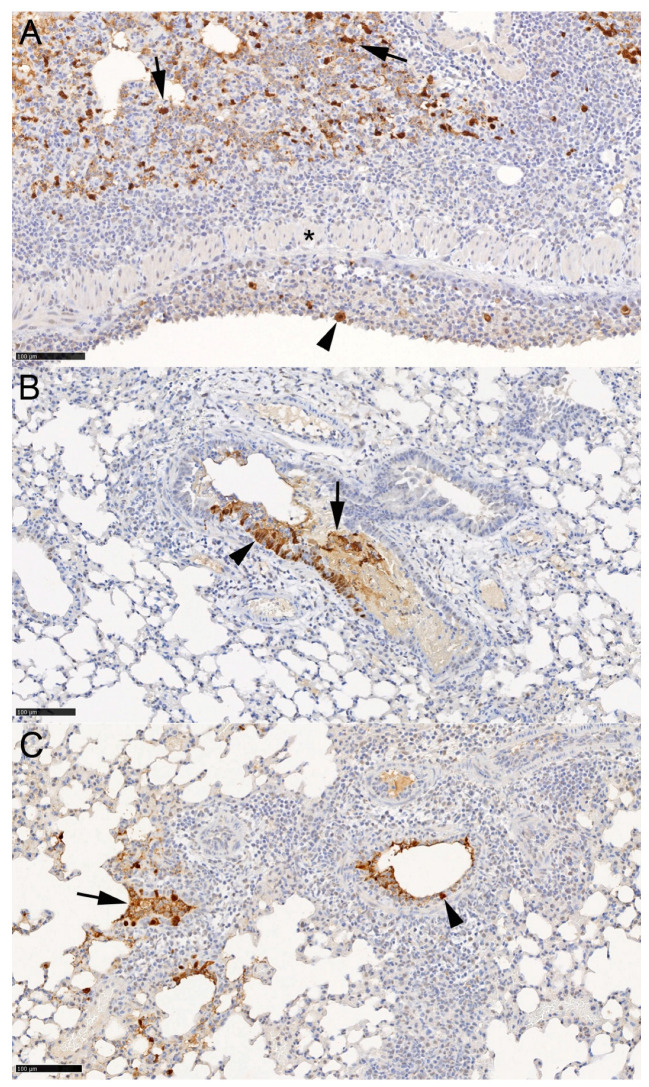
Viral antigen expression in the lungs of mice challenged with IAV, treated with ESE and examined at day 5 post-infection. Detailed individual animal data are provided in Supplementary Table S3. (**A**) Animal treated with PBS (#1.6). Bronchiole with the necrosis of epithelial cells and IAV antigen expression in sloughed-off degenerate cells (arrowhead). Extensive viral antigen expression in alveolar epithelial cells and macrophages (arrows) in an adjacent parenchymal area with changes consistent with desquamative pneumonia. Asterisk: muscular layer of the bronchiolar wall. (**B**) Animal of prophylactic treatment scheme (#2.6). Bronchiole with a patch of infected, IAV antigen-positive epithelial cells (arrowhead) and positive material in the lumen (arrow). (**C**) Animal of therapeutic treatment scheme (#4.5). Small bronchioles with intact (arrowhead) and degenerated infected epithelial cells and some positive material in the lumen. Immunohistology, hematoxylin counterstain. Bars = 100 µm.

The histological and immunohistological examinations of the vehicle-treated infected mice yielded the typical changes elicited by IAV X31 at this time point [27,28], representing necrotic bronchitis/bronchiolitis and multifocal acute desquamative pneumonia, with virus infection of both respiratory and alveolar epithelial cells and virus antigen in macrophages (Figure 10A and Figure 11A). Both the prophylactic and therapeutic treatment cohorts in the majority exhibited the same changes, though less extensively (Figure 10B,D), with evidence of free virus admixed with fluid in the lumen of bronchioles (Figure 11B,C). In contrast, virus antigen was not detected in the lungs of the animals subjected to the time of infection treatment regime. Most lungs showed no or only minimal inflammatory changes (Figure 10C); however, in one animal (#3.3), there was evidence of focal hyperplasia of the bronchiolar epithelium and a moderate (pyo)granulomatous pneumonia, suggesting that the lung had been infected. Overall, the findings suggest that pulmonary infection is blocked when ESE is applied at the time of infection. Detailed information on individual animals is provided in Supplemental Table S3.

### 3.9. Estimation and Characterisation of Phenolic Content

ESE was isolated by a hydroethanolic extraction of *Ascophyllum nodosum*, producing a crude compound rich in polysaccharides and phlorotannins, followed by the enrichment in phenolics, including phlorotannins, using solid phase extraction, which has been shown and characterised in the paper by Allwood et al. [26]. When expressed as a ratio of total carbohydrate to total phenol content, the crude extract was 3.71, while the bound (ESE) was 0.13 (Supplementary Figure S4). This shows a clear enrichment in phenol content in the bound fraction, with the remaining carbohydrate in the bound sample consisting of laminarin. This was confirmed by LC-MS analysis and analysis by high-performance anion exchange chromatography. No other monosaccharides were noted and there was no evidence for the presence of sialic acids.

The phenolic content of ESE was determined using the Folin–Ciocalteu method using a phloroglucinol standard curve and is expressed as phloroglucinol equivalents (PGE). The estimated phenolic content from the crude sample was estimated to be 155.18 PGE mg/g of dry extract, which increased substantially following enrichment in ESE to 629.47 PGE mg/g of dry extract, affording an increase in efficacy against IAV. The phlorotannins present have been characterised and confirmed previously using LC-MS [26].

## 4. Discussion

This study focused on a seaweed extract isolated from the seaweed *Ascophyllum nodosum* and its potential use as a novel antiviral agent derived from a renewable source. ESE inhibited the three strains of IAV tested most effectively when combined with the virus first. This interaction was not reversed by serial dilution and appears to cause protein aggregation, something previously seen with plant-derived tannins [30]. In contrast, the ESE pre-treatment of MDCKs did not reduce virus titres, suggesting that ESE may not interact with cells directly. The ultrastructural examination provided further evidence of this interaction between ESE and IAV, while FACS analysis using fluorescently labelled virus deduced that ESE inhibits infection by blocking virus–cell binding and therefore entry.

Interestingly, ESE appears to have more than one mode of action. The addition of ESE to cells following virus cell entry reduced viral titres and protected cells from virus-induced cell death. A time of addition assay over one replication cycle indicated that replication was not being inhibited due to the high intracellular viral load but rather the release of infectious virus. This was confirmed by the ability of ESE to inhibit NA activity in the three IAV strains, the activity of which is utilised by IAV to facilitate progeny virus release from the cell surface through the cleavage of sialic acid [10,11]. Although the inhibition of NA activity has been shown with fucoidan, a polysaccharide present in *Ascophyllum nodosum*, the enrichment of ESE reduced the polysaccharide to total phenol ratio content from 3.71 to 0.13 and afforded an increase in efficacy, indicating that the phenolic content is responsible for this antiviral activity [18,26]. Furthermore, ESE appears to reduce virus internalisation following cell binding.

The therapeutic potential of ESE was explored by utilising human 3-D airway models grown at the air–liquid interface. These displayed reduced virus titres when ESE was added to the apical surface before or following infection. Using a murine model of IAV infection, we could show that intranasally applied ESE is well tolerated when applied in a low amount of fluid rather than being instilled in a larger volume that immediately reaches the alveoli and induces a mild granulomatous reaction. The administration of ESE in mice starting at the time of infection or 3 h after infection caused a significant reduction in viral load in the lungs. It was lowest in the former cohort, from which infectious virus could not be isolated from the lungs at all. Recent work has shown that *Ascophyllum nodosum* possesses antiradical activity, something that may have contributed to the efficacy of ESE in reducing viral load and lung inflammation alongside the antiviral mechanisms of ESE described in this paper [22]. Interestingly, viral load appeared higher in the nasal tissue for the therapeutic and prophylactically treated cohorts, suggesting that ESE could be forming a protective barrier in the nasal tissue, reducing the amount of virus reaching the lungs, indicating potential for the nasal application of ESE. The effects of ESE could be compounded with the vehicle in which it is delivered, increasing retention time in the nasal cavity with a thixotropic thickener, for example, or the addition of other antiviral seaweed-derived compounds such as fucoidan, while potential synergistic effects with known influenza antivirals should be explored further.

## 5. Conclusions

ESE displays anti-IAV activity in vivo and in vitro, preventing virus–cell binding and inhibiting the release of progeny virus by targeting viral neuraminidase activity. Given the nature of the inhibition of virus–cell binding, it is possible that ESE will display antiviral efficacy against other respiratory viruses and should be investigated further. ESE displays broad-spectrum anti-IAV activity and may have the potential to be developed into a nasal spray for the prophylactic or therapeutic treatment of influenza infections.

## Figures and Tables

**Table 1:** ESE IC50 values.

Virus	Subtype	ESE IC50 (μg/mL)
PR8	H1N1	12.8
X31	H3N2	0.5
Eng195	H1N1	3.2

## Data Availability

The original contributions presented in this study are included in the article/supplementary material. Further inquiries can be directed to the corresponding author.

## Supplementary Materials

The following supporting information can be downloaded at: https://www.mdpi.com/article/10.3390/v16121919/s1, Figure S1: Cytotoxicity of ESE on MDCK and Hela cells analysed by MTS assay; Figure S2: Tolerability study of weight loss to determine pathological effects of ESE in mice. Figure S3: Tolerability study of histological changes to determine the potential pathological effects of ESE in mice. Figure S4: Recovery of sugars and total phenolic content. Table S1: Weight loss in IAV-infected mice treated with ESE, statistical analysis. Table S2: Tolerability study to determine the potential pathological effects of ESE in mice. Table S3: Study on the effect of ESE on the IAV infection of mice.

## References

[1] Lefkowitz E.J., Dempsey D.M., Hendrickson R.C., Orton R.J., Siddell S.G., Smith D.B. (2018). Virus taxonomy: The database of the International Committee on Taxonomy of Viruses (ICTV). Nucleic Acids Res..

[2] Johnson N.P.A.S., Mueller J. (2002). Updating the Accounts: Global Mortality of the 1918-1920 ‘Spanish’ Influenza Pandemic. Bull. Hist. Med..

[3] Taubenberger J.K., Morens D.M. (2010). Influenza: The once and future pandemic. Public Health Rep..

[4] Bouvier N.M., Palese P. (2008). The biology of influenza viruses. Vaccine.

[5] Chen Z., Bancej C., Lee L., Champredon D. (2022). Antigenic drift and epidemiological severity of seasonal influenza in Canada. Sci. Rep..

[6] Van de Sandt C.E., Kreijtz J.H., Rimmelzwaan G.F. (2012). Evasion of influenza a viruses from innate and adaptive immune responses. Viruses.

[7] Hay A.J., Wolstenholme A.J., Skehel J.J., Smith M.H. (1985). The molecular basis of the specific anti-influenza action of amantadine. EMBO J..

[8] Pielak R.M., Chou J.J. (2011). Influenza M2 proton channels. NIH Public Access.

[9] von Itzstein M. (2007). The war against influenza: Discovery and development of sialidase inhibitors. Nat. Rev. Drug Discov..

[10] McAuley J.L., Gilbertson B.P., Trifkovic S., Brown L.E., McKimm-Breschkin J.L. (2019). Influenza virus neuraminidase structure and functions. Front. Microbiol..

[11] Cohen M., Zhang X.-Q., Senaati H.P., Chen H.-W., Varki N.M., Schooley R.T., Gagneux P. (2013). Influenza A penetrates host mucus by cleaving sialic acids with neuraminidase. Virol. J..

[12] Matrosovich M.N., Matrosovich T.Y., Gray T., Roberts N.A., Klenk H.-D. (2004). Neuraminidase Is Important for the Initiation of Influenza Virus Infection in Human Airway Epithelium. J. Virol..

[13] Lampejo T. (2020). Influenza and antiviral resistance: An overview. Eur. J. Clin. Microbiol. Infect. Dis..

[14] Hurt A.C., Ho H.T., Barr I. (2006). Resistance to anti-influenza drugs: Adamantanes and neuraminidase inhibitors. Expert Rev. Anti Infect. Ther..

[15] Hussain M., Galvin H.D., Haw T.Y., Nutsford A.N., Husain M. (2017). Drug resistance in influenza a virus: The epidemiology and management. Infect. Drug Resist..

[16] Reis J.G., Cadamuro R.D., Cabral A.C., da Silva I.T., Rodríguez-Lázaro D., Fongaro G. (2021). Broad Spectrum Algae Compounds Against Viruses. Front. Microbiol..

[17] Usov A.I. (2011). Polysaccharides of the red algae. Advances in Carbohydrate Chemistry and Biochemistry.

[18] Wang W., Wu J., Zhang X., Hao C., Zhao X., Jiao G., Shan X., Tai W., Yu G. (2016). Inhibition of Influenza A Virus Infection by Fucoidan Targeting Viral Neuraminidase and Cellular EGFR Pathway OPEN. Sci. Rep..

[19] Ali A., Ahmed S. (2019). Carrageenans: Structure, Properties and Applications. Marine Polysaccharides.

[20] Grassauer A., Weinmuellner R., Meier C., Pretsch A., Prieschl-Grassauer E., Unger H. (2008). Iota-Carrageenan is a potent inhibitor of rhinovirus infection. Virol. J..

[21] Leibbrandt A., Meier C., König-Schuster M., Weinmüllner R., Kalthoff D., Pflugfelder B., Graf P., Frank-Gehrke B., Beer M., Fazekas T. (2010). Iota-Carrageenan Is a Potent Inhibitor of Influenza A Virus Infection. PLoS ONE.

[22] Obluchinskaya E.D., Pozharitskaya O.N., Gorshenina E.V., Daurtseva A.V., Flisyuk E.V., Generalova Y.E., Terninko I.I., Shikov A.N. (2024). *Ascophyllum nodosum* (Linnaeus) Le Jolis from Arctic: Its Biochemical Composition, Antiradical Potential, and Human Health Risk. Mar. Drugs.

[23] Echave J., Lourenço-Lopes C., Cassani L., Fraga-Corral M., Garcia-Perez P., Otero P., Carreira-Casais A., Perez-Gregorio R., Baamonde S., Saa F.F. Evidence and Perspectives on the Use of Phlorotannins as Novel Antibiotics and Therapeutic Natural Molecules. Proceedings of the 2nd International Electronic Conference on Antibiotics—Drugs for Superbugs: Antibiotic Discovery, Modes of Action and Mechanisms of Resistance.

[24] Dutot M., Fagon R., Hemon M., Rat P. (2012). Antioxidant, Anti-inflammatory, and Anti-senescence Activities of a Phlorotannin-Rich Natural Extract from Brown Seaweed *Ascophyllum nodosum*. Appl. Biochem. Biotechnol..

[25] Artan M., Li Y., Karadeniz F., Lee S.-H., Kim M.-M., Kim S.-K. (2008). Anti-HIV-1 activity of phloroglucinol derivative, 6,6′-bieckol, from Ecklonia cava. Bioorganic Med. Chem..

[26] Allwood J.W., Evans H., Austin C., Mcdougall G.J., Austin C.A. (2020). Extraction, Enrichment, and LC-MS n-Based Characterization of Phlorotannins and Related Phenolics from the Brown Seaweed, *Ascophyllum nodosum*. Mar. Drugs.

[27] Clark J.J., Penrice-Randal R., Sharma P., Dong X., Pennington S.H., Marriott A.E., Colombo S., Davidson A., Williamson M.K., Matthews D.A. (2024). Sequential Infection with Influenza A Virus Followed by Severe Acute Respiratory Syndrome Coronavirus 2 (SARS-CoV-2) Leads to More Severe Disease and Encephalitis in a Mouse Model of COVID-19. Viruses.

[28] Akram K.M., Moyo N., Leeming G., Bingle L., Jasim S., Hussain S., Schorlemmer A., Kipar A., Digard P., Tripp R. (2018). An innate defense peptide BPIFA1/SPLUNC1 restricts influenza A virus infection. Mucosal Immunol..

[29] Seehusen F., Clark J.J., Sharma P., Bentley E.G., Kirby A., Subramaniam K., Wunderlin-Giuliani S., Hughes G.L., Patterson E.I., Michael B.D. (2022). Neuroinvasion and Neurotropism by SARS-CoV-2 Variants in the K18-hACE2 Mouse. Viruses.

[30] Ueda K., Kawabata R., Irie T., Nakai Y., Tohya Y. (2013). Inactivation of Pathogenic Viruses by Plant-Derived Tannins: Strong Effects of Extracts from Persimmon (Diospyros kaki) on a Broad Range of Viruses. PLoS ONE.

[31] Haque S., Whittaker M.R., McIntosh M.P., Pouton C.W., Kaminskas L.M. (2016). Disposition and safety of inhaled biodegradable nanomedicines: Opportunities and challenges. Nanomedicine.

