# Supplementary material for: Phlorotannin-Rich Ascophyllum nodosum Seaweed Extract Inhibits Influenza Infection
Source: Viruses. 2024 Dec 15;16(12):1919. doi: 10.3390/v16121919 (PMC11680388; doi:10.3390/v16121919)
Supplement: Supplementary file 1 [file viruses-16-01919-s001.zip › viruses-3331010-supplementary.pdf]

Supplementary material

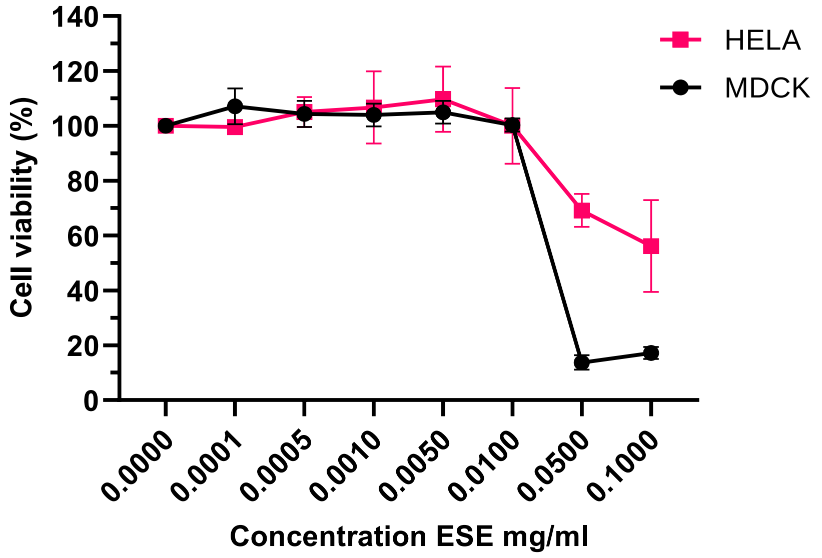

Supplementary Figure S1. Cytotoxicity of ESE on MDCK and Hela cells analysed my MTS assay. Cell viability given as a proportion of untreated control. Cell viability below 90% was determined as displaying cytotoxicity. Data represented as mean ± SEM of three independent experiments.

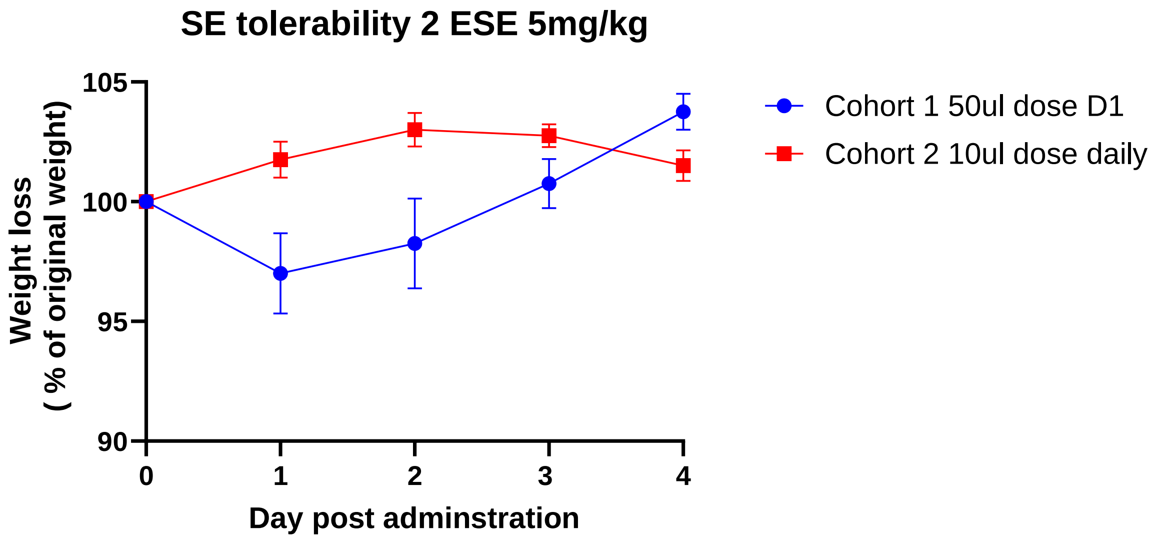

Supplementary Figure S2. Tolerability study weight loss to determine the potential pathological effects of ESE in mice. Female C57Bl/6 mice, aged 6-8 weeks, were treated with ESE at a dose of 5 mg/kg either once (in 50 µl PBS) or daily (in 10 µl PBS) for 5 consecutive days. Weight curves. Data represented as mean ± SEM (n=4).

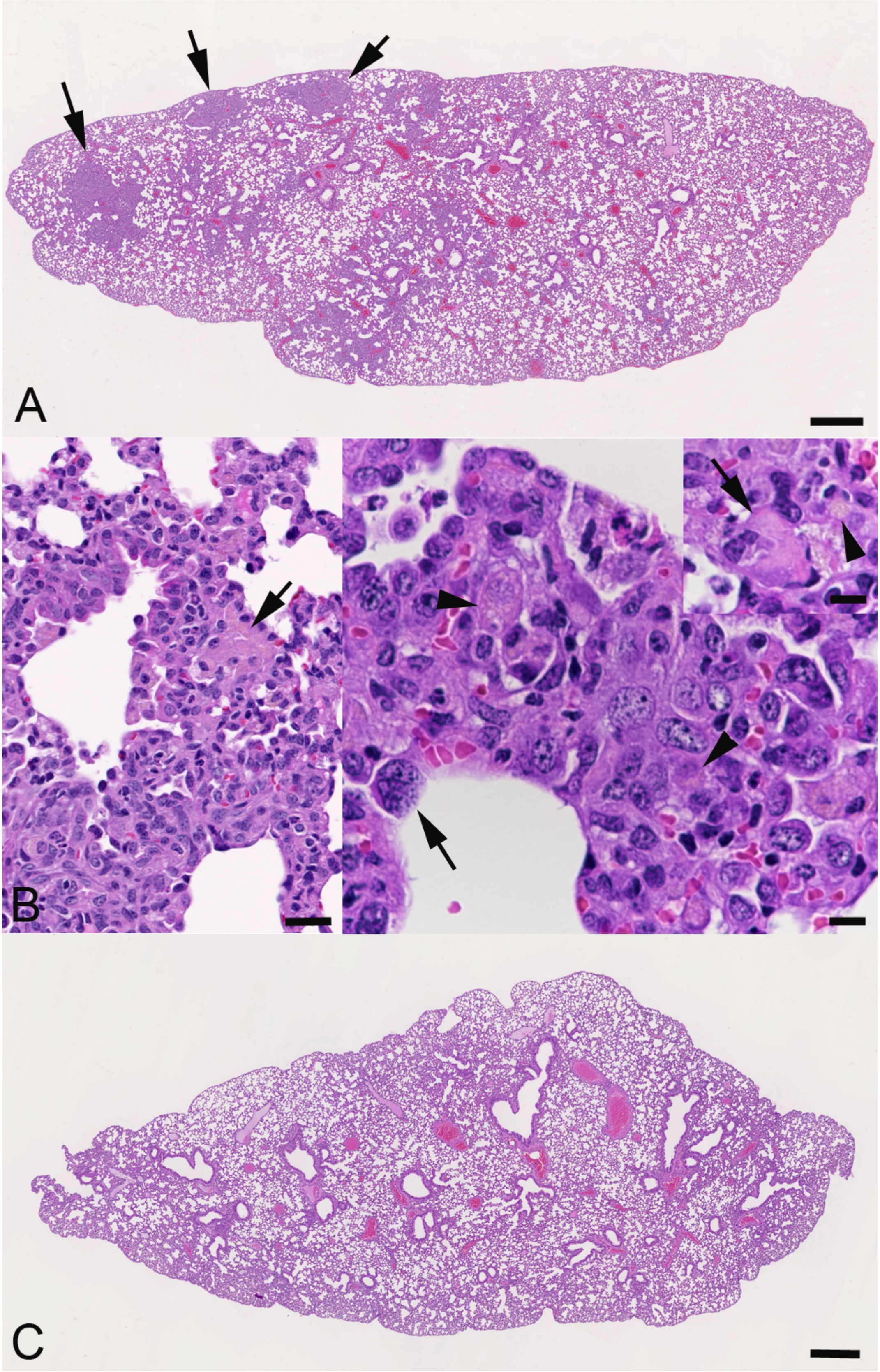

Supplementary Figure S3. Tolerability study histological changes to determine the potential pathological effects of ESE in mice. Female C57Bl/6 mice, aged 6-8 weeks, were treated with ESE at a dose of 5 mg/kg either once (in 50 µl PBS) or daily (in 10 µl PBS) for 5 consecutive days. Histological features in the lungs (detailed descriptions are provided in Supplementary Table S1). **A, B**. Animals treated with one dose in 50 µl PBS. **A.** Animal 1.1. There are several random granulomatous infiltrates

(arrows). **B.** Closer view of granulomatous infiltrates, with focal aggregate of macrophages containing amorphous eosinophilic and yellowish material (left image: arrow; animal 1.3) and individual vacuolated macrophages that contain granular, slightly yellowish material (right image incl. inset: arrowheads) and that are partly oliogonucleated (arrows) (animal 1.4). **C.** Animal treated with 4 daily doses in 10 µl PBS (animal 2.3). The lung parenchyma is unaltered. HE stain, bars = 500 µm (A, C), 50 µm (B: left image) and 10 µm (B: right image and inset).

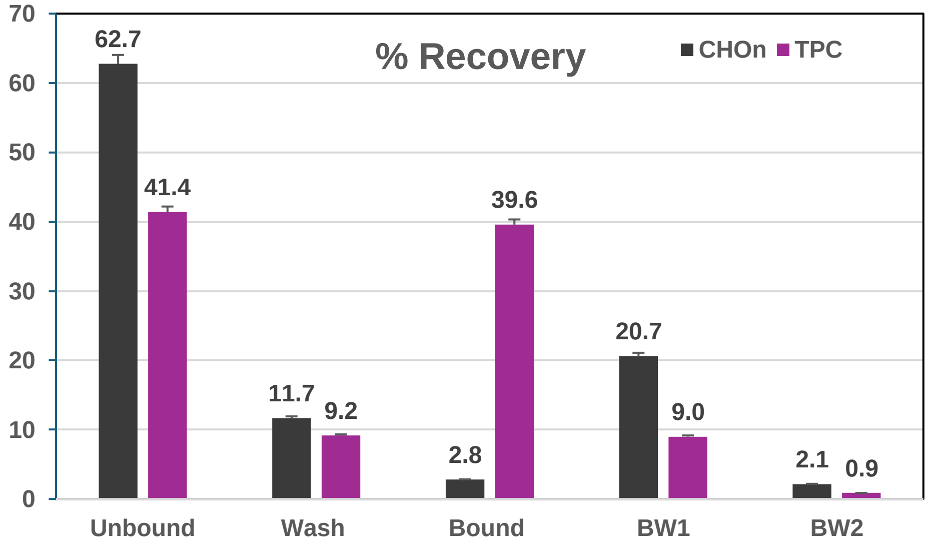

Supplementary Figure S4. Recovery of sugars and total phenolic content (TPC) expressed as a proportion of the total material in the crude sample applied to the solid phase extraction units, including washes and bound washes (BW). The bound sample (ESE) is enriched in phenolics, including phlorotannins, and this is shown and characterised in the paper Allwood et al [22]. A clear enrichment in carbohydrate in the unbound fraction and phenol content in the bound (ESE) fraction is seen. In personal communication with Dr Gordon J. McDougall of the James Hutton Institute, the remaining carbohydrate in the bound sample was mainly made up of laminarin. This was confirmed by LC-MS analysis and supported by high performance anion exchange chromatography. There was evidence for alginate in the crude and unbound samples but none in the bound fraction. Acid hydrolysis of the bound sample produced mainly glucose and mannitol from laminarin. No other monosaccharides were noted and there was no evidence for the presence of sialic acids. Furthermore, we can find no literature evidence for the presence of sialic acids.

Supplementary table S1: Weight loss in IAV infected mice treated with ESE, statistical difference. Data was compared using a repeated measures two-way ANOVA (Bonferroni post-test).

Day 1	Summary	Adjusted P Value
Vehicle vs. Prophylactic	****	<0.0001
Vehicle vs. Time of Infection	ns	0.4247
Vehicle vs. Therapeutic	*	0.0164
Day 2		
Vehicle vs. Prophylactic	**	0.0014
Vehicle vs. Time of Infection	ns	0.0825
Vehicle vs. Therapeutic	*	0.0173

Day 3		
Vehicle vs. Prophylactic	ns	0.1056
Vehicle vs. Time of Infection	ns	>0.9999
Vehicle vs. Therapeutic	ns	0.9933
Day 4		
Vehicle vs. Prophylactic	ns	0.3499
Vehicle vs. Time of Infection	ns	0.9685
Vehicle vs. Therapeutic	ns	>0.9999
Day 5		
Vehicle vs. Prophylactic	ns	>0.9999
Vehicle vs. Time of Infection	ns	0.6966
Vehicle vs. Therapeutic	ns	>0.9999

Supplementary Table S2. Tolerability study to determine the potential pathological effects of ESE in mice. Female C57Bl/6 mice, aged 6-8 weeks, were treated with ESE at a dose of 5 mg/kg either once (in 50 µl PBS) or daily (in 10 µl PBS) for 5 consecutive days, and culled on day 5. A full histological examination was undertaken on all major tissues/organs.

**Animal, treatment**	**Histological findings**
1,1 [1 x 50 µl]	**Brain, C1, eyes**: NHAIR
	**Heart**: NHAIR
	**Respiratory organs:**<br>**Trachea**: NHAIR<br>**Lung**: focal areas with a few large alveolar macrophages in lumen and activated type II pneumocytes; focal loose granulomatous infiltrates (macrophages; also in alveolar walls); a few pv leukocyte aggregates (NL, macrophages, LC)
	**Alimentary tract:**<br>**Salivary glands, tongue, oesophagus, stomach, SI, LI**: NHAIR<br>**Liver, pancreas**: NHAIR
	**Liver, pancreas**: NHAIR
	**Urinary tract (kidneys)**: NHAIR
	**Endocrine system (pituitary gland, thyroid glands, adrenal glands)**: NHAIR
	**Reproductory organs (uterus, ovaries)**: NHAIR
	**Haemolymphatic tissues:**<br>**Spleen**: mod sized primary/sec follicles and T cell zones, cell rich red pulp; **MLN, BLN, mand LN**: indistinct follicles and T cell zones, mod cellulatity; **thymus**: NHAIR; **BM**: cell rich, high haematopoietic activity
	**Skeletal muscles**: NHAIR
1,2 [1 x 50 µl]	**Brain, eyes**: NHAIR
	**Heart**: NHAIR
	**Respiratory organs:**<br>**Lung**: a few small focal (peribronchial) granulomatous infiltrates; a few pv leukocyte aggregates (NL, macrophages, LC)
	**Alimentary tract:**<br>**Salivary glands, tongue, oesophagus, stomach, SI, LI**: NHAIR<br>**Liver, pancreas**: NHAIR
	**Urinary tract (kidneys, urinary bladder)**: NHAIR
	**Endocrine system (adrenal glands)**: NHAIR

	**Reproductory organs (uterus, ovaries)**: NHAIR
	**Haemolymphatic tissues:**<br>**Spleen**: mod sized primary/sec follicles and T cell zones, cell rich red pulp; **MLN, BLN**: indistinct follicles and T cell zones, mod cellulatity; **thymus**: NHAIR; **BM**: cell rich, high haematopoietic activity
	**Skeletal muscles**: NHAIR
	**Skin**: NHAIR
1,3<br>[1 x 50 µl]	**Brain, eyes**: NHAIR
	**Heart**: NHAIR
	**Respiratory organs:**<br>**Trachea**: NHAIR<br>**Lung**: a few small focal (peribronchial) granulomatous infiltrates; a few pv leukocyte aggregates (NL, macrophages, LC)
	**Alimentary tract:**<br>**Salivary glands, tongue, oesophagus, stomach, SI, LI**: NHAIR<br>**Liver, pancreas**: NHAIR
	**Liver, pancreas**: NHAIR
	**Urinary tract (kidneys, urinary bladder)**: NHAIR
	**Endocrine system (pituitary gland, thyroid glands, adrenal glands)**: NHAIR
	**Reproductory organs (uterus, ovaries)**: NHAIR
	**Haemolymphatic tissues:**<br>**Spleen**: mod sized primary/sec follicles and T cell zones, cell rich red pulp; **BLN, mand LN**: no distinct follicles, mod cellularity; **thymus**: NHAIR; **BM**: cell rich, high haematopoietic activity
	**Skeletal muscles**: NHAIR
1,4<br>[1 x 50 µl]	**Brain, C1, eyes**: NHAIR
	**Heart**: NHAIR
	**Respiratory organs:**<br>**Trachea**: NHAIR<br>**Lung**: multifocal granulomatous infiltrates (macrophages; also in alveolar walls), in larger lesions with embedded NL aggregates; a few pv leukocyte aggregates (NL, macrophages, LC)
	**Alimentary tract:**<br>**Salivary glands, oesophagus, stomach, SI, LI**: NHAIR<br>**Liver, pancreas**: NHAIR
	**Urinary tract (kidneys)**: NHAIR
	**Endocrine system (pituitary gland, adrenal glands)**: NHAIR
	**Pituitary gland, adrenal glands**: NHAIR
	**Reproductory organs (uterus, ovaries)**: NHAIR
	**Haemolymphatic tissues:**<br>**Spleen**: mod sized primary/sec follicles and T cell zones, cell rich red pulp; **MLN, BLN**: indistinct follicles and T cell zones, mod cellulatity; **thymus**: NHAIR; **BM**: cell rich, high haematopoietic activity
	**Skeletal muscles, femorotibial joint**: NHAIR
	**Skin**: NHAIR
2,1<br>[4 x 10 µ]	**Brain, C1, eyes**: NHAIR
	**Heart**: NHAIR
	**Respiratory organs:**<br>**Trachea**: NHAIR<br>**Lung**: mild focal pv mixed cellular infiltration
	**Alimentary tract:**<br>**Salivary glands, oesophagus, stomach, SI, LI**: NHAIR<br>**Liver, pancreas**: NHAIR
	**Urinary tract (kidneys, urinary bladder)**: NHAIR
	**Endocrine system (pituitary gland, adrenal glands)**: NHAIR
	**Reproductory organs (uterus, ovaries)**: NHAIR
	**Haemolymphatic tissues:**

	**Spleen**: mod sized primary/sec follicles and T cell zones, cell rich red pulp; **MLN, mand LN**: indistinct follicles and T cell zones, mod cellularity; **thymus**: NHAIR; **BM**: cell rich, high haematopoietic activity
	**Skeletal muscles, femorotibial joint**: NHAIR
	**Skin**: NHAIR
2,2 [4 x 10 µ]	**Brain, C1, eyes**: NHAIR
	**Heart**: NHAIR
	**Respiratory organs:** **Trachea**: NHAIR **Lung**: one small artery with mild focal leukocyte rolling and pv accumulation
	**Alimentary tract:** **Salivary glands, oesophagus, stomach, SI, LI**: NHAIR **Liver, pancreas**: NHAIR
	**Urinary tract (kidneys)**: mild focal mononuclear interstitial infiltration in one kidney
	**Endocrine system (pituitary gland, thyroid glands, adrenal glands)**: NHAIR
	**Reproductory organs (uterus, ovaries)**: NHAIR
	**Haemolymphatic tissues:** **Spleen**: mod sized primary/sec follicles and T cell zones, cell rich red pulp; **MLN**: small section; **thymus**: NHAIR; **BM**: cell rich, high haematopoietic activity
	**Skeletal muscles, femorotibial joint**: NHAIR
	**Skin**: NHAIR
2,3 [4 x 10 µ]	**Brain, C1, eyes**: NHAIR
	**Heart**: NHAIR
	**Respiratory organs (trachea, lung)**: NHAIR
	**Alimentary tract:** **Salivary glands, tongue, oesophagus, stomach, SI, LI**: NHAIR **Liver, pancreas**: NHAIR
	**Urinary tract (kidneys, urinary bladder)**: NHAIR
	**Endocrine system (adrenal glands)**: NHAIR
	**Reproductory organs (uterus, ovaries)**: NHAIR
	**Haemolymphatic tissues:** **Spleen**: mod sized primary/sec follicles and T cell zones, cell rich red pulp; **MLN, mand LN**: indistinct follicles and T cell zones, mod cellulatity; **thymus**: NHAIR; **BM**: cell rich, high haematopoietic activity
	**Skeletal muscles**: NHAIR
	**Skin**: NHAIR
2,4 [4 x 10 µ]	**Brain, C1, eyes**: NHAIR
	**Heart**: NHAIR
	**Respiratory organs (trachea, lung)**: NHAIR
	**Alimentary tract:** **Salivary glands, tongue, oesophagus, stomach, SI, LI**: NHAIR **Liver, pancreas**: NHAIR
	**Urinary tract (kidneys)**: NHAIR
	**Endocrine system (pituitary gland, thyroid glands, adrenal glands)**: NHAIR
	**Reproductory organs (uterus, ovaries)**: NHAIR
	**Haemolymphatic tissues:** **Spleen**: mod sized primary/sec follicles and T cell zones, cell rich red pulp; **MLN**: indistinct follicles and T cell zones, mod cellulatity; **thymus**: NHAIR; **BM**: cell rich, high haematopoietic activity
	**Skeletal muscles, femorotibial joint**: NHAIR
	**Skin**: NHAIR

Legend: BLN – bronchial lymph node; C1 – spinal cord at level of C1; LC – lymphocytes; LI – large intestine; mand LN – mandibular lymph node; MLN – mesenteric lymph node; mod – moderate(ly); NHAIR – no histological abnormality is recognised; NL – neutrophilic leukocytes (neutrophils); pv - perivascular; SI – small intestine

Supplementary Table S3. Study on the effect of ESE on IAV infection of mice. Female C57Bl/6 mice, aged 6-8 weeks, were infected with a sublethal dose of IAV (cohorts 1-4) and treated with PBS at 3 hpi, then daily (animals 1.1 to 1.6) or with ESE at a dose of 5 mg/kg in 10 µl PBS at 2 h pre-infection, 3 hpi, then daily (animals 2-1 to 2-6), at time of infection, 3 hpi, then daily (animals 3.1 to 3.5) or at 3 hpi, then daily (animals 4.1 to 4.6). Mock-infected mice that received PBS at 3 hpi, then daily (animals 5.1 to 5.3) served as controls. All mice were euthanised at day 5 post infection. A histological examination and immunohistology for IAV antigen was undertaken on the lung.

Animal No	Histological findings, immunohistology for viral antigen
1.1	**Lung**: many bronchioles with partly flattened, partly necrotic BEC, partly with individual necrotic bronchial/-iolar BEC, with LC dominated bronchial and pb infiltration, abundant degenerate cells, debris and NL in lumen; adjacent focal parenchymal areas with desquamation of AM/type II pn, sometimes necrotic cells, activated type II pn, some NL and LC; vasculitis and pv LC-dominated mononuclear infiltration<br>**vAg**: numerous bronchioles with mod number of pos BEC (intact and degen, individual or patches; in lumen) and AEC (and macrophages) in parenchymal infiltrates adjacent to affected bronchioles
1.2	**Lung**: bronchus with focal area of BEC necrosis and LC dominated bronchial and pb infiltration, degenerate cells, debris and NL in lumen; two small focal parenchymal areas with desquamation of AM/type II pn, activated type II pn, some NL and LC<br>**vAg**: bronchus with large patch of pos BEC (intact and degen, individual or patches; in lumen), one bronchiole with a few individual pos BEC; AEC (and macrophages) in parenchymal infiltrates
1.3	**Lung**: bronchus with focal BEC necrosis, loss and debris in lumen, with LC dominated bronchial and pb infiltration; focal parenchymal area with desquamation of AM/type II pn, some necrotic cells, activated type II pn, some NL and LC; mild vasculitis and pv LC-dominated mononuclear infiltration<br>**vAg**: bronchus with almost diffuse pos BEC; several bronchioles with large patches of pos BEC; AEC (and macrophages) mainly in parenchymal infiltrate
1.4	**Lung**: one large parenchymal area with embedded bronchiole exhibiting BEC necrosis, loss and debris in lumen, with LC dominated bronchial and pb infiltration; parenchyma with desquamation of AM/type II pn, some necrotic cells, activated type II pn, some NL and LC; vasculitis and pv LC-dominated mononuclear infiltration<br>**vAg**: in one large area bronchioles with variable mount of pos BEC (intact and degen); pos AEC (and macrophages) mainly in extensive parenchymal infiltrate
1.5	**Lung**: one large parenchymal area with embedded bronchiole exhibiting complete BEC necrosis, loss and debris in lumen, with LC dominated bronchial and pb infiltration; parenchyma with desquamation of AM/type II pn, some necrotic cells, activated type II pn, some NL and LC; vasculitis and pv LC-dominated mononuclear infiltration<br>**vAg**: in one large area bronchioles with variable amount of pos BEC (intact and degen); pos AEC (and macrophages) mainly in extensive parenchymal infiltrate
1.6	**Lung**: bronchus and several bronchioles with (partly) necrotic BEC, with LC dominated bronchial and pb infiltration, abundant degen cells, debris and NL in lumen; adjacent focal parenchymal areas with desquamation of AM/type II pn,

	some necrotic cells, activated type II pn, some NL and LC; mild vasculitis and pv LC-dominated mononuclear infiltration<br>**vAg**: several bronchioles with mod number of pos BEC (intact and degen, individual or patches; in lumen); AEC (and macrophages) in parenchymal infiltrates adjacent to affected bronchioles
2.1	**Lung**: a few bronchioles with complete necrosis and loss of BEC, filled with abundant degen cells, debris and proteinaceous material; with LC dominated bronchial and pb infiltration; one to several adjacent focal granulomatous infiltrates with embedded NL (similar also in alveoli); also small parenchymal areas with desquamation of AM/type II pn, some necrotic cells, activated type II pn, some NL and LC; mild vasculitis and pv LC-dominated mononuclear infiltration<br>**vAg**: in BEC and cell free in material in lumen of bronchi
2.2	**Lung**: two larger and a few small nodular focal pb granulomatous infiltrates with embedded individual NL and aggregates of LC; large bronchiole with activated, partly hyperplastic BEC and attached clumped proteinaceous material<br>**vAg**: individual pos BEC in several bronchioles, also cell free in material in lumen of bronchi
2.3	**Lung**: NHAIR<br>**vAg**: one bronchiole with one pos BEC; one small area with a few pos AEC
2.4	**Lung**: mod focal pv LC-dominated mixed cellular infiltration and adjacent parenchymal area with proteinaceous material and degen cells in alveolar lumina and mixed inflammatory infiltrate; small focal granulomatous parenchymal infiltrate adjacent to small bronchiole<br>**vAg**: neg
2.5	**Lung**: one large bronchiole with focal pb LC dominated infiltration and adjacent focal consolidated area with desquamation of AM/type II pn, some necrotic cells, activated type II pn, some NL and LC<br>**vAg**: one bronchiole with one pos BEC; focal lesion with numerous pos AEC (and macrophages) within and immediately adjacent to looser consolidated areas
2.6	**Lung**: mild multifocal pv LC-dominated infiltration, one pv parenchymal area with degen cells in alveolar lumina and mixed inflammatory infiltrate; one bronchiole with proteinaceous material and degen cells in lumen<br>**vAg**: one bronchiole with a patch of pos BEC, also cell free in material in lumen of bronchi
3.1	**Lung**: NHAIR<br>**vAg**: neg
3.3	**Lung**: mod multifocal pv LC-dominated, partly mixed cellular infiltration; one vessel with marked focal NL infiltration and adjacent parenchymal area with marked NL infiltration; several nodular focal granulomatous parenchymal infiltrates adjacent to bronchioles, partly NL dominated; a few bronchioles with mild to mod BEC hyperplasia and some debris in lumen<br>**vAg**: neg
3.4	**Lung**: NHAIR<br>**vAg**: neg
3.5	**Lung**: multifocal (nodular) granulomatous parenchymal infiltrates adjacent to bronchioles, with variable proportions of NL; a few bronchioles with mild BEC hyperplasia and some debris in lumen<br>**vAg**: neg
3.6	**Lung**: NHAIR, apart from rare very small focal granulamatous infltrates<br>**vAg**: neg
4.1	**Lung**: focal area with mod pb and pv LC infiltration; focal consolidated area with desquamation of AM/type II pn, some necrotic cells, activated type II pn, some NL and LC<br>**vAg**: one large area with several bronchioles with mod number of pos BEC (intact and degen, individual or patches; in lumen, with some clumped cell free antigen)

	and AEC (and macrophages) in parenchymal infiltrates adjacent to affected bronchioles
4.2	**Lung**: NHAIR, apart from very mild focal LC aggregates<br>**vAg**: one bronchiole with one pos BEC
4.3	**Lung**: focal area with mod pb and pv LC infiltration; adjacent focal area with desquamation of AM/type II pn, some necrotic cells, activated type II pn, some NL and LC, alveolar oedema<br>**vAg**: two bronchioles with large patch of pos BEC; pos AEC and macrophages in alveoli in the periphery of focal parenchymal area with inflammatory infiltration
4.4	**Lung**: NHAIR<br>**vAg**: neg
4.5	**Lung**: bronchus with mod to marked pb LC-dominated (and focally NL dominated) infiltration, some degen sloughed off BEC and abundant NL in lumen<br>**vAg**: bronchus with several individual pos BEC and some degen pos BEC in lumen (with debris)
4.6	**Lung**: focal area with mod pb and pv LC infiltration (with some NL); focal area with desquamation of AM/type II pn, some necrotic cells, activated type II pn, some NL and LC, and leukocyte recruitment<br>**vAg**: focal area with a few pos BEC in some bronchioles and a few adjacent pos AEC (and macrophages), several pos AEC and macrophages in focal parenchymal area with inflammatory infiltration
5.1	**Lung**: NHAIR; **vAg**: neg
5.2	**Lung**: NHAIR; **vAg**: neg
5.3	**Lung**: NHAIR; **vAg**: neg

Legend: AEC – alveolar epithelial cells; AM – alveolar macrophages; BEC – bronchiolar epithelial cells; degen – degenerate; LC – lymphocytes; neg – negative; NHAIR – no histological abnormality is recognised; NL – neutrophilic leukocytes (neutrophils); pn – pneumocytes; pos – positive; pv – perivascular; vAg – viral antigen

Cohort 1: PBS 3 hpi, then daily
Cohort 2: ESE 5mg/kg, 2 h pre-inf, 3 hpi, then daily
Cohort 3: 5mg/kg, time of inf, 3 hpi, then daily
Cohort 4: 5 mg/kg, 3 hpi, then daily
Cohort 5: Uninfected; PBS, 3 hpi, then daily
